# Heterointerface‐Modulated Synthetic Synapses Exhibiting Complex Multiscale Plasticity

**DOI:** 10.1002/advs.202417237

**Published:** 2025-05-20

**Authors:** Xingji Liu, Yao Ni, Zujun Wang, Sunfu Wei, Xiao′en Chen, Jingjie Lin, Lu Liu, Boyang Yu, Yue Yu, Dengyun Lei, Yayi Chen, Jianfeng Zhang, Jing Qi, Wei Zhong, Yuan Liu

**Affiliations:** ^1^ School of Integrated Circuits Guangdong University of Technology Guangzhou 510006 China; ^2^ National Key Laboratory of Intense Pulsed Irradiation Simulation and Effect Northwest Institute of Nuclear Technology Xi’an 710024 China; ^3^ School of Material Science and Engineering University of Jinan Jinan 250022 China; ^4^ School of Materials and Energy Lanzhou University Lanzhou 730000 China; ^5^ Department of Land Surveying and Geo‐Informatics The Hong Kong Polytechnic University Kowloon Hong Kong 999077 China

**Keywords:** bio‐inspired cryptographic applications, dual‐neurotransmitters, lateral modulation, spatiotemporal properties, synaptic transistor

## Abstract

An asymmetric dual‐gate heterointerface‐regulated artificial synapse (HRAS) is developed, utilizing a main gate with distinct ion concentrations and a lateral gate to receive synaptic pulses, and through dielectric coupling and ionic effects, formed indium tin zinc oxide (ITZO) dual‐interface channels that allow precise control over channel charge, thereby simulating multi‐level coordinated actions of dual‐neurotransmitters. The lateral modulation of the lateral gate significantly regulates ionic effects, achieving the intricate interplay among lateral inhibition/enhancement and short‐/long‐term plasticity at a multi‐level scale for the first time. This interplay enables the HRAS device to simulate frequency‐dependent image filtering and spike number‐dependent dynamic visual persistence. By combining temporal synaptic inputs with lateral modulation, HRAS harnesses spatiotemporal properties for bio‐inspired cryptographic applications, offering a versatile device‐level platform for secure information processing. Furthermore, a novel dual‐gate input neural network architecture based on HRAS has been proposed, which aids in weight update and demonstrates enhanced recognition capabilities in neural network tasks, highlighting its role in bio‐inspired computing.

## Graphical abstract

An asymmetric dual‐gate heterointerface‐regulated artificial synapse (HRAS) is developed, employing distinct ion concentrations and dielectric coupling to form precise dual‐interface channels. Through lateral modulation, HRAS realizes, for the first time, the intricate interplay of lateral inhibition/enhancement and multi‐level short‐ and long‐term plasticity, offering new possibilities for advanced neuromorphic functionalities.

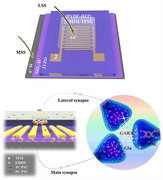

## 1 Introduction

In neuromorphic computing, replicating the intricate neural networks and processing mechanisms of the brain is essential.^[^ ^1^, ^2^, ^3^, ^4^, ^5^, ^6^, ^7^ ^]^ The biological brain can select appropriate functions and effectively respond to complex external environments, relying on its intricate neural conduction processes, where the synergistic action of various neurotransmitters is essential for responding to diverse stimulus signals.^[^ ^8^, ^9^, ^10^, ^11^ ^]^ This composite neural conduction aids in constructing complex neural circuits, thereby allowing the brain to integrate information adaptively.^[^ ^12^, ^13^ ^]^ For instance, lateral synaptic regulation, which includes both lateral excitation and lateral inhibition mechanisms, the former may enhance the activity of specific neurons,^[^ ^14^, ^15^ ^]^ while the latter can highlight the edges of stimuli, increasing contrast.^[^ ^16^, ^17^ ^]^ They play a vital role in balancing excitatory activities, ensuring precise signal transmission, promoting selective information processing, and dynamically modulating the connectivity of neural networks.

Although the development of synaptic transistors has achieved some breakthroughs in mimicking the complex synaptic structures of the human brain, most synaptic devices currently can only simulate the release of a single neurotransmitter.^[^ ^18^, ^19^, ^20^, ^21^ ^]^ Consequently, these devices are typically unable to fully replicate the lateral modulation functions within neural networks, especially the multidimensional regulation that switches from lateral inhibition to lateral potentiation.^[^ ^22^, ^23^ ^]^ A select few synaptic devices, utilizing bipolar semiconductors or P/N heterostructures, can emulate two neurotransmitter types via electrons and holes.^[^ ^24^, ^25^ ^]^ However, the mobility of the two types of carriers usually differs significantly due to the inherent polarity mismatch of the charge transport channels. These results in the current response dominated by the weaker party being suppressed, thus failing to exhibit its true synaptic characteristics, posing challenges in simulating neuron interactions and regulatory mechanisms. Inspired by this, the creation of diverse nanoscale electron‐ion coupled interfaces utilizing a single semiconductor material to achieve charge‐based lateral modulation is anticipated to facilitate artificial synapses featuring multi‐level, dual‐neurotransmitter‐coordinated plasticity.

We have developed an innovative asymmetric dual‐gate heterointerface‐regulated artificial synapse (HRAS). It simulates lateral regulation in neuronal interactions, addressing the limitations of existing three‐terminal and two‐terminal artificial synapse devices in replicating complex biological mechanisms.^[^ ^26^, ^27^, ^28^, ^29^ ^]^ The HRAS utilizes a main gate with varying ion concentrations and a lateral gate for capturing synaptic pulses. Through electronic coupling and ionic effects, it forms ITZO dual‐interface channels, allowing precise control over channel charge and simulating the multi‐level coordinated actions of dual‐neurotransmitters. The lateral modulation of the lateral gate in the HRAS significantly regulates ionic effects, firstly achieving the ability to switch between lateral inhibition/enhancement and short/long‐term plasticity under different lateral synaptic stimulations. This mechanism enables the HRAS device to process frequency‐dependent image high‐pass filtering and mimick the mechanisms of dynamic visual persistence. Furthermore, by combining temporal synaptic inputs with lateral modulation, HRAS harnesses spatiotemporal properties for bio‐inspired cryptographic applications, providing a versatile device‐level platform for secure information processing. We further proposed a novel dual‐gate input neural network architecture, which not only aids in weight tuning but also demonstrates enhanced recognition capabilities in neural network tasks. This new architecture highlights the role of HRAS in bio‐inspired computing, showcasing its potential in the development of more efficient algorithms for image processing and object recognition. It is expected to become a component of neural network computing hardware, marking a significant advancement in the field of artificial neural systems.

## 2 Results and Discussion

**Figure 1:**
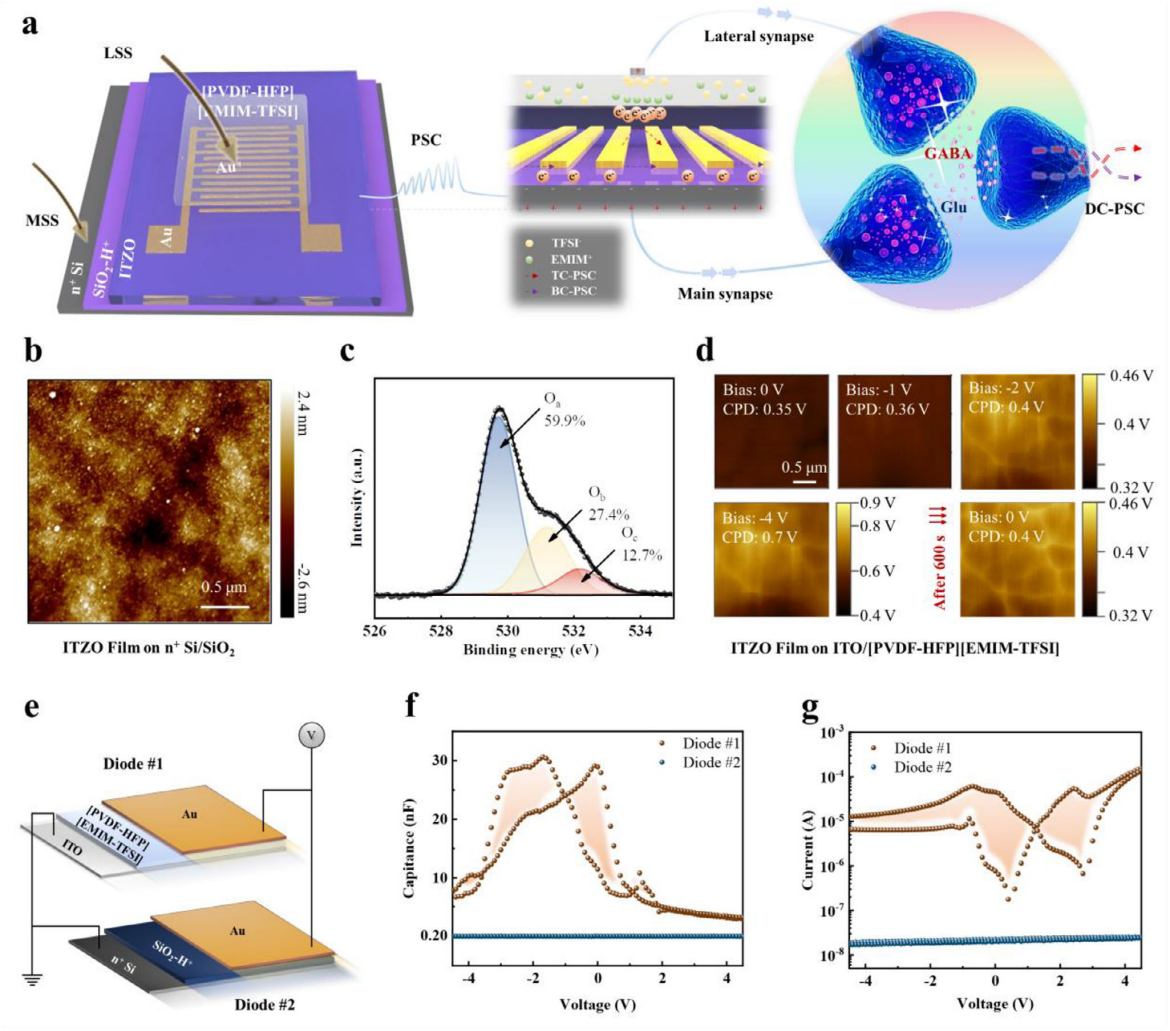
a) Schematic of HRAS and biological synapses for multi‐level intertwined transmission of different neurotransmitters (i.e., Glu and GABA). b) AFM images of an ITZO layer on a SiO_2_ substrate. c) XPS survey spectra of O 1s of a ITZO film. d) KPFM measurement of the surface potential of ITZO in a glass/ITO/[PVDF‐HFP][EMIM‐TFSI] ion gel/ITZO structure under tip voltages of 0, −1, −2, and −4 V. e) Illustration of two diode devices with the architectures ITO/[PVDF‐HFP][EMIM‐TFSI] ion gel/Au (diode #1) and n^+^Si/SiO_2_‐H^+^/Au (diode #2). f) C‐V characteristic curves of diode #1 and diode #2. g) I‐V characteristic curves of diode #1 and diode #2.

A heterointerface‐regulated synaptic transistor (HRAS) with an asymmetric dual‐gate structure was designed to mimic the lateral synaptic regulation (**Figure** **1**a). A large‐sized, heavily doped n‐type Si layer, coated with surface‐adsorbed H^+^ on silicon dioxide (SiO_2_‐H^+^), serves as the primary gate for receiving main synaptic spikes (MSS). Contrasting this, a small square electrode located on an ion gel containing 1‐ethyl‐3‐methylimidazolium (EMIM^+^) cations and bis(trifluoromethanesulfonyl)imide (TFSI^−^) anions serves as the lateral gate for capturing lateral synaptic spikes (LSS), which exhibits excellent performance in terms of ionic transport efficiency, low‐voltage stimulation responsiveness, and solvent resistance. HRAS employs a dual‐channel dynamic modulation mechanism based on a 30 nm‐thick ITZO layer, synergistically regulated by the MSS and LSS to dynamically simulate the multi‐level coordinated actions between excitatory glutamic acid (Glu) and inhibitory γ‐aminobutyric acid (GABA) neurotransmitters in biological synapses. When a positive voltage is applied to MSS, the dielectric coupling effect of the SiO_2_ layer and trace H^+^ ions trigger the formation of a weak electric double layer (EDL) at the bottom interface, activating the lower conductive channel and generating output current, corresponding to the excitatory response induced by Glu release in biological synapses. A positive LSS drives cation accumulation at the ITZO top interface, forming a strong EDL to enhance current (simulating the synergistic enhancement of Glu due to reduced GABA concentration). A negative LSS suppresses the activation of H^+^ ions at the bottom channel interface and impedes charge carrier transport through vertical spatial electric field coupling, thereby reducing the current output. This phenomenon is analogous to the antagonistic effect of elevated GABA concentration on Glu. The resulting dual‐channel postsynaptic current (DC‐PSC), emerging through the source‐drain electrodes, replicates the dynamic interplay of excitatory signal amplification and inhibitory regulation in biological synapses.

An atomic force microscope (AFM) image indicates that the ITZO film deposited on SiO_2_ via the sputtering process has a compact, smooth interface with an average roughness *R*_a_ of 0.54 nm (Figure 1b). The band gap Eg of ITZO was determined to be 3.12 eV through measurements and calculations using UV–vis spectroscopy (Figure S1, Supporting Information). The chemical bonding state of the metal‐oxide is characterized by X‐ray photoelectron spectroscopy (XPS) (Figure 1c). The O 1s spectrum fitted with Gaussian‐Lorentz can be decomposed into three peaks, O_a_, O_b_, and O_c_, corresponding to lattice‐bound oxygen, defect oxygen in the lattice, and chemisorbed oxygen, respectively. Among these, O_b_ and O_c_ are crucial for the ion‐induced plasticity control characteristics in synaptic devices.^[^ ^30^ ^]^ The permeation and accumulation of ions significantly influence the memory characteristics of HRAS. Using Kelvin Probe Force Microscopy (KPFM), we investigated the surface potential of ITZO thin films within a Class/ITO/[PVDF‐HFP][EMIM‐TFSI] ion gel/ITZO structure. Initially, the average surface potential (CPD) of the ITZO film was measured at 0.35 V. When top‐gate biases of −1 and −2 V were applied, the CPD increased slightly to 0.36 and 0.4 V, respectively, and surged to 0.7 at −4 V. Six hundred seconds post‐bias removal, the CPD stabilized at 0.4 V. These results confirm that substantial biases promote EMIM^+^ permeation and accumulation within the ITZO film.

The capacitance‐voltage *C*‐*V* and current‐voltage *I*‐*V* curves of the diodes based on ion gel (diode #1) and SiO_2_ (diode #2) are tested to evaluate the ability to regulate channel charge of the asymmetrical dual‐gates (Figure 1e–g). Diode #1 exhibits a significant hysteresis effect, while the hysteresis effect in diode #2 is less pronounced, indicating that the charge storage characteristics dominated by the ion gel are much greater than SiO_2_. Therefore, by designing the differential working areas based on SiO_2_‐H^+^ and the ion gel interfaces rationally, the output current intensity of the dual channel can be matched, enabling a precise match of output current intensities and a multi‐level intertwined plasticity.

**Figure 2:**
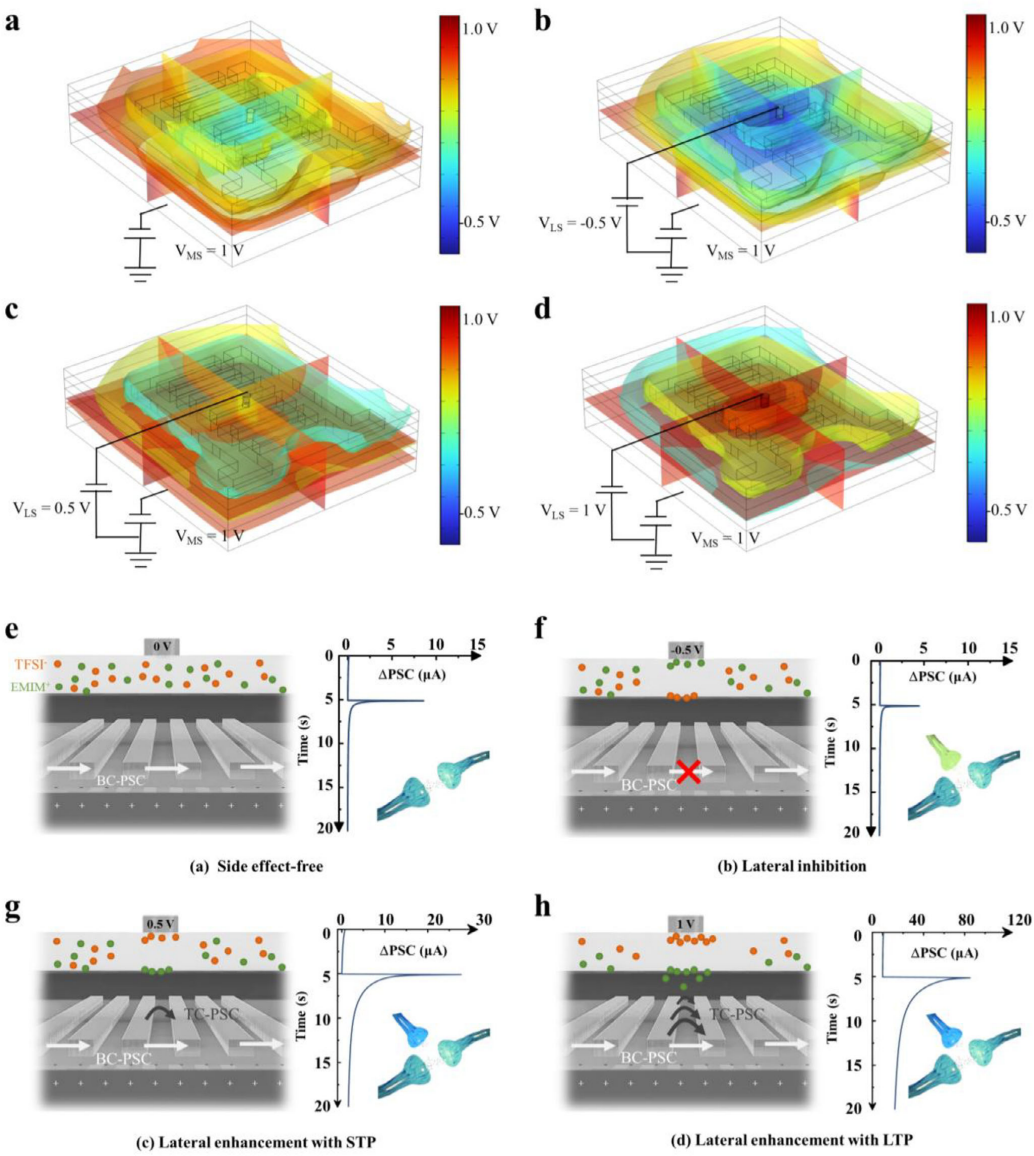
Electric‐field distribution in the device structure simulated at programming processes of a) *V*_MS_ = 1 V, b) *V*_MS_ = 1 V and *V*_LS_ = −0.5 V, c) *V*_MS_ = 1 V and *V*_LS_ = 0.5 V, and d) *V*_MS_ = 1 V and *V*_LS_ = 1 V. e) Side‐view device sketches illustrating ion distribution and charge transport under MSS of 1 V; Corresponding ΔPSC (PSC – *I*_ini_; *I*_ini_: current before applied spikes) exhibiting a lateral modulation‐free response. f) Side‐view device sketches illustrating ion distribution and charge transport under MSS of 1 V and LSS of −0.5 V; Corresponding ΔPSC exhibiting a lateral inhibition. g) Side‐view device sketches illustrating ion distribution and charge transport under MSS of 1 V and LSS of 0.5 V; Corresponding ΔPSC exhibiting a lateral enhancement with STP. h) Side‐view device sketches illustrating ion distribution and charge transport under MSS of 1 V and LSS of 1 V; Corresponding ΔPSC exhibiting a lateral enhancement with LTP.

To elucidate the modulation effects of asymmetric dual gates on the dual‐interface channel of HRAS, finite element simulations using COMSOL were employed to visualize the internal electric field distribution under varying LSS conditions. When a 1 V *V*_MS_ is applied exclusively to the main gate, a uniformly distributed positive potential emerges at the bottom channel interface due to electric field coupling through the SiO_2_ dielectric (**Figure** **2**a). Upon the application of an additional −0.5 V *V*_LS_ at the lateral gate, the more robust electric field eclipses the coupled potential initiated by the main gate, leading to a negative potential across the entire vertical space beneath the lateral gate coverage; while the region not covered by the lateral gate experiences a gradual transition from a negative potential at the center to a positive potential at the periphery (Figure 2b). In contrast, a *V*_LS_ of +0.5 V induces a localized positive shift in potential at the top channel interface, primarily within the ion gel covered region (Figure 2c). Further increasing VLS to 1 V amplifies this effect, producing an even stronger positive potential across the top interface (Figure 2d). Additionally, the conductance test results of HRAS demonstrate significant modulation by MSS and LSS (Figure S2, Supporting Information).

We further delve into the response mechanisms of HRAS upon various external stimuli by examining the distribution of ions and charges. When a 1 V MSS is applied exclusively to the main gate in the absence of lateral modulation, a minor activation of H^+^ at the bottom interface occurs, establishing a weak electric double‐layer (EDL) with the ITZO bottom interface. The EDL effect, in conjunction with the dielectric coupling effect, induces the transport of charge carriers at the bottom interface, thereby simulating the excitatory bottom‐channel postsynaptic current (BC‐PSC) predominantly mediated by Glu (Figure 2e). Upon the application of an additional −0.5 V LSS to the lateral gate, the negative electric field coupling through the ion gel inhibits both the activation of H^+^ and the transport of charge carriers across the entire vertical space. Consequently, the excitatory BC‐PSC and charge storage capacity are diminished, mimicking the lateral inhibition state synergistically induced by GABA (Figure 2f). Conversely, when an additional 0.5 V LSS is applied to the lateral gate, the initially disordered distribution of positive and negative ions within the ion gel is reorganized under the coupled electric field. A substantial accumulation of EMIM^+^ at the ITZO top interface occurs, resulting in the formation of a potent EDL and the activation of the top interface to serve as an auxiliary conduit for electronic charge carrier transport, thereby eliciting a top‐channel PSC (TP‐PSC) (Figure 2g). Although the charge storage capacity is marginally enhanced, the accumulated cations at the interface rapidly return to their initial positions upon pulse removal, leading to a swift decay of the PSC. This scenario simulates the enhancement of Glu‐induced postsynaptic currents under conditions of reduced GABA release, while the memory state remains characterized by GABA‐sensitive short‐term synaptic plasticity (STP). Upon increasing the LSS to 1 V, cations accelerate toward the top interface of ITZO, significantly enhancing the electrical double layer (EDL) effect. Concurrently, with certain EMIM^+^ penetrating the top interface of ITZO. Unlike the reversible migration observed under lower voltage conditions, these cations remain at the top interface for an extended period after the pulse, thereby sustaining charge carrier transport and effectively emulating a Glu release‐dominated long‐term plasticity (LTP) state (Figure 2h). The relaxation time constant (*τ*) was determined to characterize synaptic plasticity, where *τ* < 1 s corresponds to STP. Upon increasing the LSS voltage from −0.5 to 1 V, the value of *τ* exhibited a significant enhancement from 67 ms to 1.388 s, effectively crossing the temporal threshold from STP to LTP (Figure S3, Supporting Information). This result implicates the lateral gate mechanism in regulating the short‐long term memory transition.^[^ ^31^, ^32^, ^33^, ^34^, ^35^, ^36^ ^]^ Unlike conventional artificial synapses that mimic the synergistic or competitive mechanisms of two neurotransmitters through heterojunctions or p‐i‐n structures (comprising two semiconductor layers), the HRAS pioneers the use of a heterointerface based on a single unipolar semiconductor channel to simulate dual‐neurotransmitter synergy. It exhibits greater sensitivity to input voltage, a broader weight modulation range (Figure S4, Supporting Information). Moreover, the HRAS is the first to modulate biological lateral synaptic behavior by adjusting voltage polarity and amplitude, thereby achieving complex interactions between lateral potentiation/inhibition and LTP/STP synaptic plasticity across multiple levels (Table S1, Supporting Information).^[^ ^12^, ^13^, ^24^, ^37^, ^38^, ^39^, ^40^, ^41^, ^42^, ^43^ ^]^

**Figure 3:**
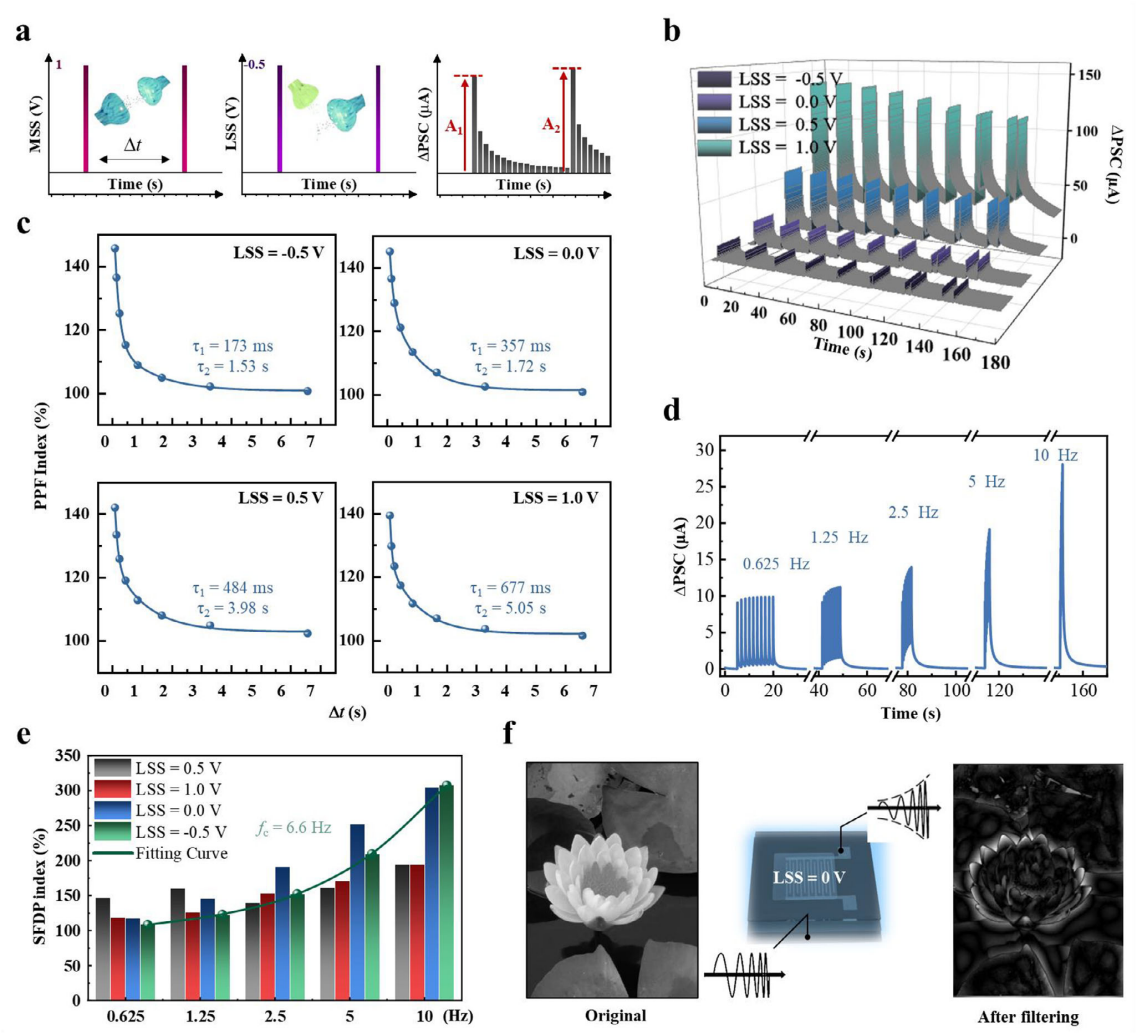
a) Synergistic modulation of PPF triggered by a pair of MSSs with an Δ*t*, and a pair of LSSs with the same Δ*t*. A_1_ and A_2_ represent the ΔPSC values at the end of the first and the subsequent spikes, respectively. b) ΔPSCs triggered by pairs of MSSs of 1 V with different Δ*t*, under the coordinated control of different LSSs. c) PPF indexes (A_2_/A_1_ × 100%) as a function of Δ*t*, under the coordinated control of different LSSs. d) ΔPSCs triggered by a series of LSSs of different frequencies, with the coordinated control of 1 V MSS. e) SFDP indexes (A_f_/A_0.625_ × 100%) triggered by a series of MSSs of different frequencies, under the coordinated control of different LSSs. f) Sharpening of an image with the high‐pass filtering function at the cut off frequency of 6.6 Hz.

Paired‐pulse facilitation (PPF), involves the rapid enhancement of synaptic efficacy and the escalation of synaptic transmission efficiency.^[^ ^44^, ^45^ ^]^ Despite its critical function in neural signal processing, there is a dearth of artificial synaptic models capable of dynamically modulating PPF. We develop for the first time the realization of dynamically adjustable PPF utilizing the lateral modulation network of HRAS. A pair of successive 1 V MSSs with varying time intervals Δ*t* were delivered to the main gate, while tunable LSSs were concurrently triggered (**Figure** **3**a). With an increase in LSS intensity, the accumulation of ions facilitating charge carrier transport at the channel interface escalates, thereby inducing a greater PSC (Figure 3b). The attenuation of the PPF index with increasing Δ*t* is well‐described by a double exponential fitting (DEF), mirroring the properties of genuine biological synapses (Figure 3c). Moreover, as the LSSs were incremented from −0.5 to 1 V, the facilitation capability exhibited flexible modulation, with the rapid relaxation time *τ*_1_ escalating from 173 to 677 ms, and the slow relaxation time *τ*_2_ from 1.53 s to 5.05 s (Method Section, Supporting Information).^[^ ^46^, ^47^ ^]^

The synaptic weight can be intuitively altered by modulating the frequency of input spikes, a process known as spike‐frequency‐dependent plasticity (SFDP).^[^ ^48^, ^49^ ^]^ PSCs in response to continuous spikes of different frequencies were recorded: as the MSS frequency increases, PSC substantially increases (Figure 3d; Figure S5, Supporting Information). This enhancement is attributed to the high‐frequency spikes intensifying the accumulation of cations and reducing the likelihood of ion back‐diffusion within the HRAS. The SFDP index under various LSS conditions is depicted as a function of presynaptic spike frequency (Figure 3e). Since the strength of the top electric double layer (EDL) is greater than that of the bottom EDL, the top EDL has a stronger response to high‐frequency pulses, causing the required LSS value for a higher SFDP index to decrease as the trigger frequency rises. At 10 Hz, the SFDP index peaks under negative LSS conditions. Overall, the SFDP index varies most with frequency at LSS = −0.5 V, offering significant advantages for time‐frequency filtering applications. This behavior suggests that high‐frequency input signals surpassing a certain cut off value, fc can pass through HRAS devices, while low‐frequency signals are markedly diminished. Figure 3f presents a schematic illustration of OST devices acting as high‐pass filters, using the processing of a lotus image as an example to emulate the filtering process: when applying *f*_c_ = 6.6 Hz for high‐pass filtering, the contour features are sharpened (Method Section, Supporting Information).

**Figure 4:**
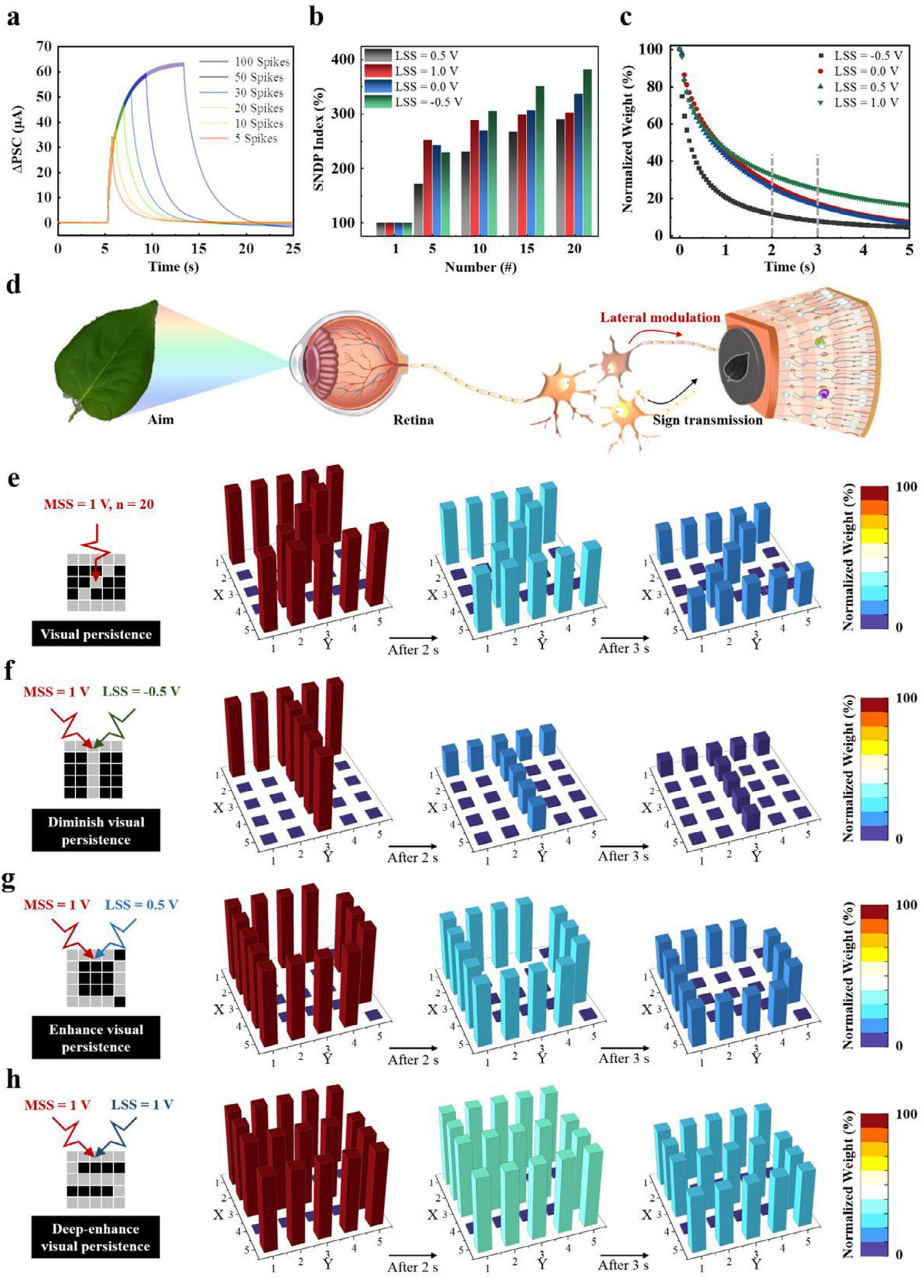
a) ΔPSCs triggered by a series of MSSs of different numbers, without the coordinated control of LSSs. b) SNDP indexes (A_n_/A_1_ × 100%) triggered by a series of MSSs of different numbers, under the coordinated control of different LSSs. c) Normalized weight retention curve after removing 20 spikes, under the coordinated control of different LSSs. d) Illustration of the visual persistence effect under different lateral modulations. A series of MSSs programmed HRAS array, emulating 5 × 5‐pixel image in e) visual persistence event without LSS, f) diminish visual persistence with LSS = −0.5 V, g) enhance visual persistence with LSS = 0.5 V, and h) deep‐enhance visual persistence with LSS = 1 V.

Repeated stimulation can elicit the additional release of neurotransmitters and modulate the synaptic connections between neurons, a phenomenon referred to as spike number‐dependent plasticity (SNDP).^[^ ^50^, ^51^ ^]^ Upon exposed to a series of 1 V MSS pulses, HRAS emulates SNDP behavior (**Figure** **4**a). As the number of spikes (*n*) increases, both the PSC and SNDP index exhibit a synchronous upward trend. The SNDP indexes under the modulation of LSS of varying intensities are also documented (Figure 4b; Figures S6–S9, Supporting Information): the SNDP index similarly increases with *n*, indicating that the EDL effect is enhanced with the accumulation of stimulation. The PSC demonstrates ideal linearity when a small LSS of ≤ 0.5 V is applied, suggesting that ionic activation at the interface remains below saturation. However, at a higher bias of 1 V LSS, the SNDP index exhibits a marked slowdown in growth after the fifth spike, accompanied by a pronounced degradation in the linear correlation between PSC and *n* (Figure 4b; Figure S10, Supporting Information).

Visual persistence effect refers to the phenomenon whereby the visual perception of briefly presented images or objects endures for a finite period.^[^ ^52^, ^53^ ^]^ During visual search tasks, individuals are required to swiftly recognize congruent visual objects and expeditiously discard the memory of searched image features to avert interference from visual data. In contrast, mental rotation tasks demand the short‐term maintenance and subsequent processing of entire images, thus requiring sustained retention of visual information. By virtue of its unique properties (Figure 4d) and demonstrated reliability (Figure S11, Supporting Information), HRAS enables precise modulation of visual persistence under varying lateral modulation conditions.

The integration with weight‐mapped imagery is exemplified by the visual representation of the “ZTDS” letters on a 5 × 5 synaptic unit grid, mimicking letter images constructed from an array of 25 pixels. Applying 20 MSSs of 1 V without lateral modulation is sufficient to rapidly adjust the weights within the target letter range, with signal weights exhibiting a decay yet persisting beyond 3 s, thus replicating the basic visual persistence effect (Figure 4e). The co‐application of negative LSSs of −0.5 V with the MSSs markedly expedites the signal decay post‐stimulation, indicative of attenuated visual persistence (Figure 4f). In contrast, the imposition of positive LSSs of 0.5 V augments visual persistence, retaining information more robustly 3 s after stimulus withdrawal than in the absence of LSSs (Figure 4g). Furthermore, elevating the LSSs to 1 V markedly intensifies the information storage capacity, exhibiting a deep‐enhanced visual persistence (Figure 4h).

The postsynaptic response is further characterized by spike‐duration dependent plasticity (SDDP) (Figures S12–S15, Supporting Information).^[^ ^54^, ^55^ ^]^ With the augmentation of spike duration *d*, both the EDL effect and the PSC are intensified. The SDDP indices modulated by LSS of varying intensities indicate that as LSS increases from −0.5 to 1 V, there is a swift saturation of cation accumulation, leading to an overall decline in the SDDP index, with a gradual enhancement in induced charge storage.

**Figure 5:**
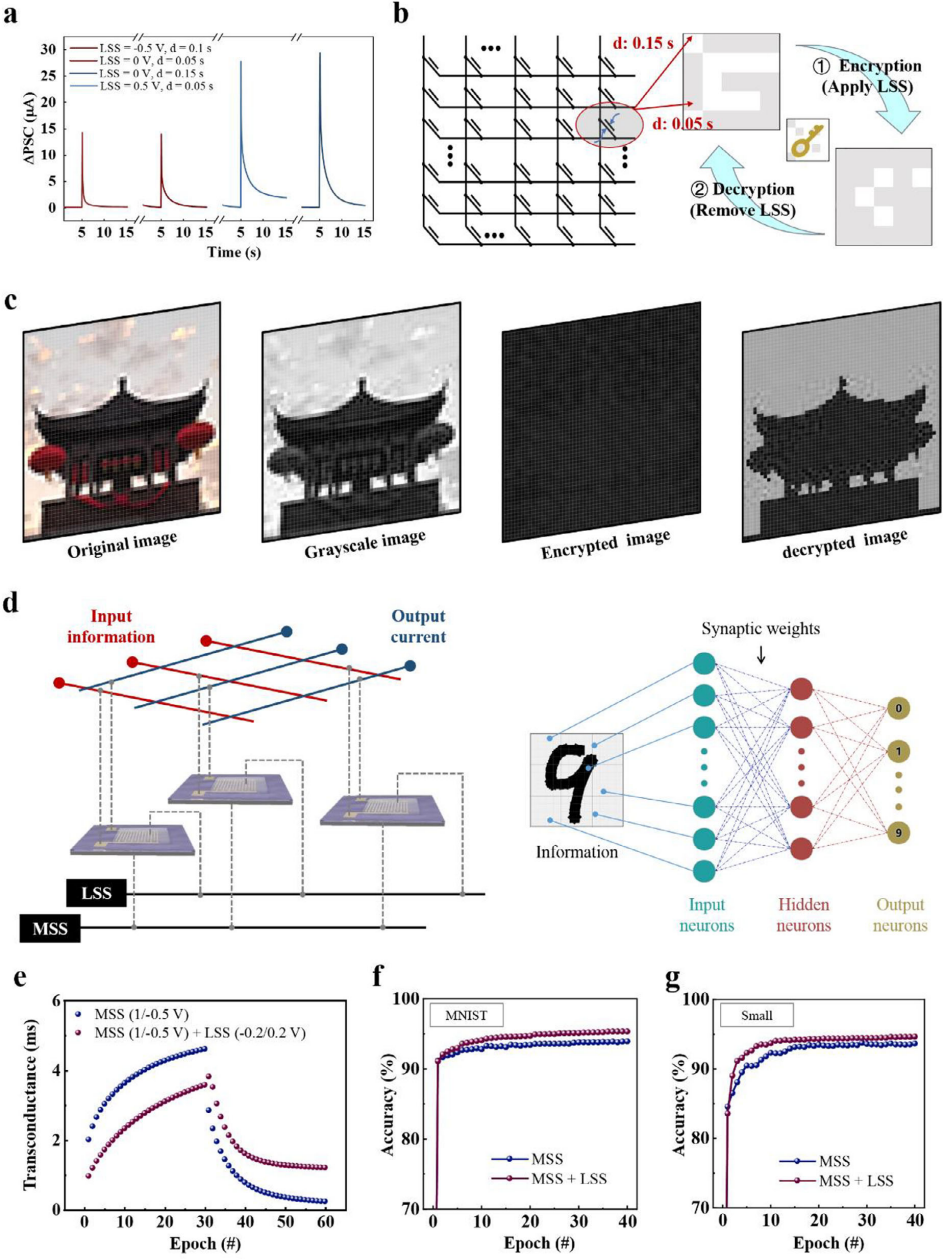
a) ΔPSCs triggered by a series of MSSs of different durations, under the coordinated control of different LSSs. b) Illustration of image encryption and decryption using HRAS array constructed with LSS as the key. c) Encrypted and decrypted images of a drum tower photo after grayscale processing controlled by LSS. d) Dual‐bar input neural network structure for image recognition of the MNIST dataset constructed by HRAS, controlled through coordinated modulation of MSS and LSS. e) Potentiation‐depression regulations of HRAS with and without LSS modulation. f) Recognition accuracy of small images with 28 × 28 pixels after the training process. g) Recognition accuracy of large images with 8 × 8 pixels after the training process.

In the absence of lateral gate activity, a 0.05 s MSS induces a minor ΔPSC of 14 µA, matching the PSC triggered by a 0.1 s MSS at LSS = −0.5 V. Similarly, a 0.15 s MSS without lateral modulation triggers a larger ΔPSC of 29 µA, equivalent to the ΔPSC triggered by a 0.1 s MSS at LSS = −0.5 V (**Figure** **5**a). It is worth noting that all of them fall within the STP category and apply to encryption operations for real‐time signal processing. A series of ΔPSC with a weak fluctuation were observed across 10 parallel control groups, indicating a promising applicability (Figure S16, Supporting Information). Employing LSS as a key auxiliary input to regulate output signal characteristics constitutes a device‐level encryption strategy, bolstering data security with a physical safeguard against breaches (Figure 5b). In an encrypted integrated array generated by HRAS devices, PSC corresponds to image grayscale values; the weaker and stronger PSCs, triggered by 0.05 and 0.15 s MSS inputs, represent background noise and significant information, respectively. Additional negative LSS inputs can mask background noise as significant information within the original matrix, while positive LSS inputs can also camouflage significant information as background noise. After the LSS is withdrawn, the jumbled image information reverts to the display of the original image, achieving decryption.

Expanding on this concept, we present an encryption and decryption process within an image matrix, utilizing lateral modulations to distinguish between background noise and significant information, actuated by pulses from the external signal port (Figure 5c). When the LSS key is engaged, the genuine image information is obscured; once the LSS key is disengaged, the genuine image information becomes apparent. This innovation integrates spatiotemporal synaptic input with lateral modulation for the first time, leveraging their spatiotemporal properties to solve the dynamic confusion encryption problem in Optoelectronic devices and the difficulty of electro‐controlled devices in processing spatiotemporal analog signals. It paves the way for bionic encryption applications. This advancement marks the first integration of temporal synaptic input with lateral modulation, promoting the development of bionic encryption applications (Table S2, Supporting Information).^[^ ^56^, ^57^, ^58^, ^59^, ^60^, ^61^, ^62^, ^63^, ^64^ ^]^

Capitalizing on the lateral modulations inherent to HRAS, a dual‐bar input neural network architecture can be engineered to bolster the precision of pattern recognition tasks. Within a crossbar matrix, each HRAS unit engages in vector‐matrix multiplication and outer‐product update operations, controlled by voltage as depicted in the circuit diagram (Figure 5d): The conductance states are orchestrated by the synergistic activation of MSS and LSS through dual‐bar inputs, multiplied with voltage signal representations along the red rows; the resultant currents are conveyed by the blue rows. Employing alternating sequences of 30 positive and negative MSSs demonstrates the tuning of conductance weights, while the integration of LSS enhances the linearity and symmetry of weight modulation, providing a significant boost for artificial neural networks that employ backpropagation (Figure 5e). Without LSS, the recognition rates for the MNIST and Small datasets stabilize at 93.19% and 92.15% after 40 epochs, respectively (Figure 5f). With LSS integration, these rates are elevated to 94.09% and 93.49%, underscoring the efficacy of the dual‐bar input modulation approach in amplifying the capabilities of artificial neural networks (Figure 5g).

## 3 Conclusion

In this research, we developed HRAS with the asymmetric dual‐gate structure to simulate lateral synaptic modulation, showcasing its capabilities in neuromorphic computing and data security. The HRAS utilizes a main gate and a lateral gate to capture synaptic spikes and form ITZO dual‐interface channels through dielectric coupling and ionic effects, mimicking the coordinated action of multiple neurotransmitters. The design allows precise control over channel charge, enabling the intertwining of multi‐level plasticity and modulating synaptic weights. By integrating spike frequency and number modulation‐induced synaptic weight modulation with lateral control, the HRAS device simulates image filtering and dynamically adjustable visual persistence. HRAS leverages spatiotemporal properties for bio‐inspired cryptographic applications by integrating temporal synaptic inputs with lateral modulation, providing a versatile device‐level platform for secure information processing. Furthermore, the dual‐gate input neural network structure based on HRAS facilitates the weight adjustment, highlighting its role in bio‐inspired computing and providing a robust framework for simulating complex neural functions and enhancing the performance of artificial neural networks.

## Conflict of Interest

The authors declare no conflict of interest.

## Supporting information



Supporting Information

## Data Availability

The data that support the findings of this study are available from the corresponding author upon reasonable request.
