# Supplementary material for: Heterointerface‐Modulated Synthetic Synapses Exhibiting Complex Multiscale Plasticity
Source: Adv Sci (Weinh). 2025 May 20;12(30):e17237. doi: 10.1002/advs.202417237 (PMC12376703; doi:10.1002/advs.202417237)
Supplement: Supplementary file 1 — Supporting Information [file ADVS-12-e17237-s001.docx]

**Supporting Information**

**Heterointerface-Modulated Synthetic Synapses Exhibiting Complex Multiscale Plasticity**

*Xingji Liu, Yao Ni*, Zujun Wang, Sunfu Wei, Xiao′en Chen, Jingjie Lin, Lu Liu, Boyang Yu, Yue Yu, Dengyun Lei, Yayi Chen, Jianfeng Zhang, Jing Qi, Wei Zhong*, and Yuan Liu**

Mr. X. Liu, Dr. Y. Ni, Mr. S. Wei, Mr. X. Chen, Ms. J. Lin, Dr. D. Lei, Dr. Y. Chen, Dr. J. Zhang, Dr. W. Zhong, and Prof. Y. Liu

School of Integrated Circuits, Guangdong University of Technology, Guangzhou 510006, PR China

Prof. Zujun Wang

National Key Laboratory of Intense Pulsed Irradiation Simulation and Effect, Northwest institute of nuclear technology, Xi^’^an 710024, China

Dr. Lu Liu

School of Material Science and Engineering, University of Jinan, Jinan 250022, China

Mr. Boyang Yu, and Prof. Jing Qi

School of Materials and Energy, Lanzhou University, Lanzhou 730000, China

Dr. Yue Yu

Department of Land Surveying and Geo-Informatics, The Hong Kong Polytechnic University, Kowloon, Hong Kong

*Corresponding author:

[niyao0723@163.com](mailto:niyao0723@163.com) and [niyao@gdut.edu.cn](mailto:niyao@gdut.edu.cn) (Dr. Y. Ni)

[zwnice@163.com](mailto:zwnice@163.com) (Dr. W. Zhong)

eeliuyuan@gdut.edu.cn (Prof. Y. Liu)

**Experimental** **Section**

***Fabrication of HRAS.*** A heavily n-doped silicon wafer (<0.02 Ω cm) with a 300 nm thick thermally oxidized SiO_2_ layer was used as the bottom gate and dielectric. Firstly, a 30 nm ITZO film was deposited as the electron transport channel at room temperature using magnetron sputtering with InSnO and ZnO targets. Subsequently, the ITZO film underwent an annealing process in an air atmosphere at 350°C for 3 hours. The source and drain Au electrodes were thermally deposited through an interdigital shadow mask (width 2000 × 11 μm; length 100 μm) onto the ITZO film. Then the ion-gel dielectric (the mass ratio between polymer Poly(vinylidenefluoride-co-hexafluoropropylene) PVDF-HFP and ionic liquid 1-ethyl-3-methylimidazolium bis(trifluoromethylsulfonyl)imide EMIM-TFSI is 1:3) layer was transferred onto the channel area. A square Au electrode with an area of 1 mm^2^ was deposited through thermal evaporation at the center of the ion gel as the top gate.

***Characterization and electronical measurements.*** AFM image of the ITZO was collected using a Bruker dimension icon microscope operated in tapping mode. The contact potential difference (CPD) distribution of ITZO thin films in an ITO/[PVDF-HFP][EMIM-TFSI]/ITZO structure was characterized using in situ Kelvin probe force microscopy (KPFM) measurements. XPS was conducted using a Thermo Scientific (ESCALAB 250Xi). The optical absorption spectra were performed using a UV-Vis spectrophotometer (Cary 5000) at room temperature. All electrical measurements were measured at room temperature under atmosphere by a Keithley 4200CS semiconductor analyzer.

***Device Simulations.*** The internal electric potential distribution in the HRAS device was modeled and simulated by using COMSOL Multi-physics under varying LSS conditions. To describe the steady-state electric fields of the device, the mathematical and physical modules with the Primary Current Distribution Interface were combined in the model. The current and voltage relationship follows Ohm's law and satisfies the current conservation equation. The initial potential was set to 0 V. In the simulation, one side of the Interdigitated shaped electrode was set to 0 V, the other side to 0.01 V, and the bottom electrode to 1 V. Finally, the potential distribution of the device was solved for the top electrode at -0.5 V, 0 V, 0.5 V, and 1 V. The neural network simulations of arrays devices for digital recognition was performed in CrossSim platform, which provided a clean python application programming interface so that different algorithms can be built upon resistive memory crossbars while modeling realistic device properties and variability. A three-layer (one hidden layer) neural network was used to execute supervised learning over the training examples after which the network accuracy was compared against the test examples in a single training epoch. The simulations took into account the device nonidealities as well as analog to digital (A/D) and digital to analog (D/A) conversion by “external electronics” that interfaced with the crossbar by sending voltages and summing currents along the rows and columns. The network simulations were carried out on a MNIST image version (28 × 28 pixels) and a Small image version (8 × 8 pixels) of handwritten digits. After training with 60000-image training set, a separate 10000-image testing set was used for recognition.

**Method Section**

***PSC-retention time fitting.*** The PSC-retention curves were fitted using a simple first-order stretched exponential function: *I* = (*I*_0_ - *I*_∞_) × exp[-(*t*/*τ* - *t*_0_/*τ*)*^β^*] + *I*_∞_; where *t*_0_ = 0 is the time that the presynaptic spike finishes, *I*_0_ is the initial PSC when the spike finishes, *I*_∞_ is the final PSC in an equilibrium state, 0 ≤ *β* ≤ 1 is a stretch index, and *τ* is the retention time.

***PPF index fitting.*** The decay of the PPF index with the increase of Δ*t* fits a DEF equation with a rapid decay and a slow decay: PPF index = 100% + *C*_1_ × exp [-(Δ*t*/*τ*_1_)] + *C*_2_ × exp [-(Δ*t*/*τ*_2_)]; where *C*_1_ (*C*_2_) and *τ*_1_ (*τ*_2_) represent the initial facilitation magnitudes and characteristic relaxation time of rapid (slow) phases, respectively.

***SFDP index fitting.*** The dependence of PSC on peak frequency was well fitted with the sigmoidal function: H(*f*) = (*a*_1_ - *a*_2_) / (1 + (*f*/*f*_c_))*^p^* + *a*_2_; where *p* is the order of the function, *f*_c_ is the cut-off frequency, *a*_1_ and *a*_2_ are the initial and final amplitude.

***Low-frequency suppression simulation.*** To simulation a low-frequency suppression filter, a grayscale image of a lotus as an example was first converted from spatial domain to frequency domain by Fourier transform. Then the matrix in the frequency domain was rearranged by moving the zero-frequency component to the center. An m × n filter template was proposed depended on the size of the generating new matrix. A high-pass filter was established by exploiting the SFDP under negative PSPs. In the filter template, *f* is the Euclidean distance between the center (*u* = 0.5 m, *v* = 0.5 n) and a specific point (*i*, *j*), as *f* = [(*i* - *u*)^2^ + (*j*- *v*)^2^]^0.5^, and *f*_c_ = 6.6 Hz is the cut-off frequency of the filter. The filtering was implemented by utilizing a matrix *G* = H(*i*, *j*) N(*i*, *j*). *G* was decentralized and transformed to a matrix in the spatial domain by inverse Fourier transform, to achieve the ultimate result.

**Supplementary Figures**


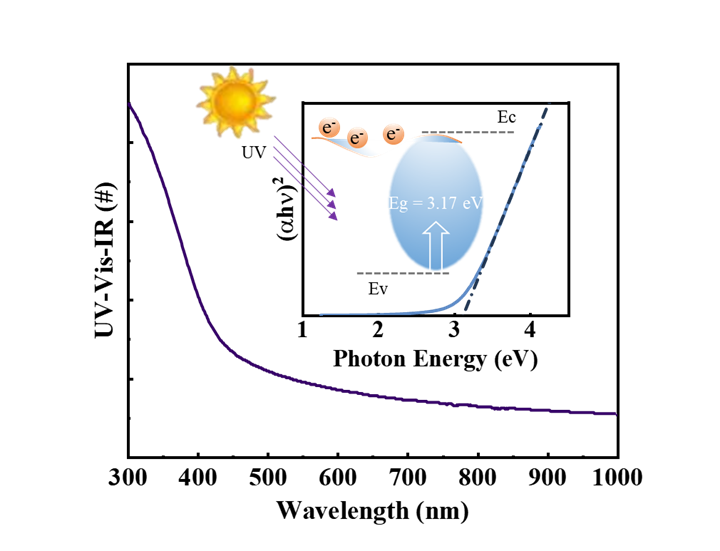


**Figure S1.** The band gap Eg of ITZO was determined to be 3.12 eV through measurements and calculations using ultraviolet-visible (UV-Vis) spectroscopy.

**
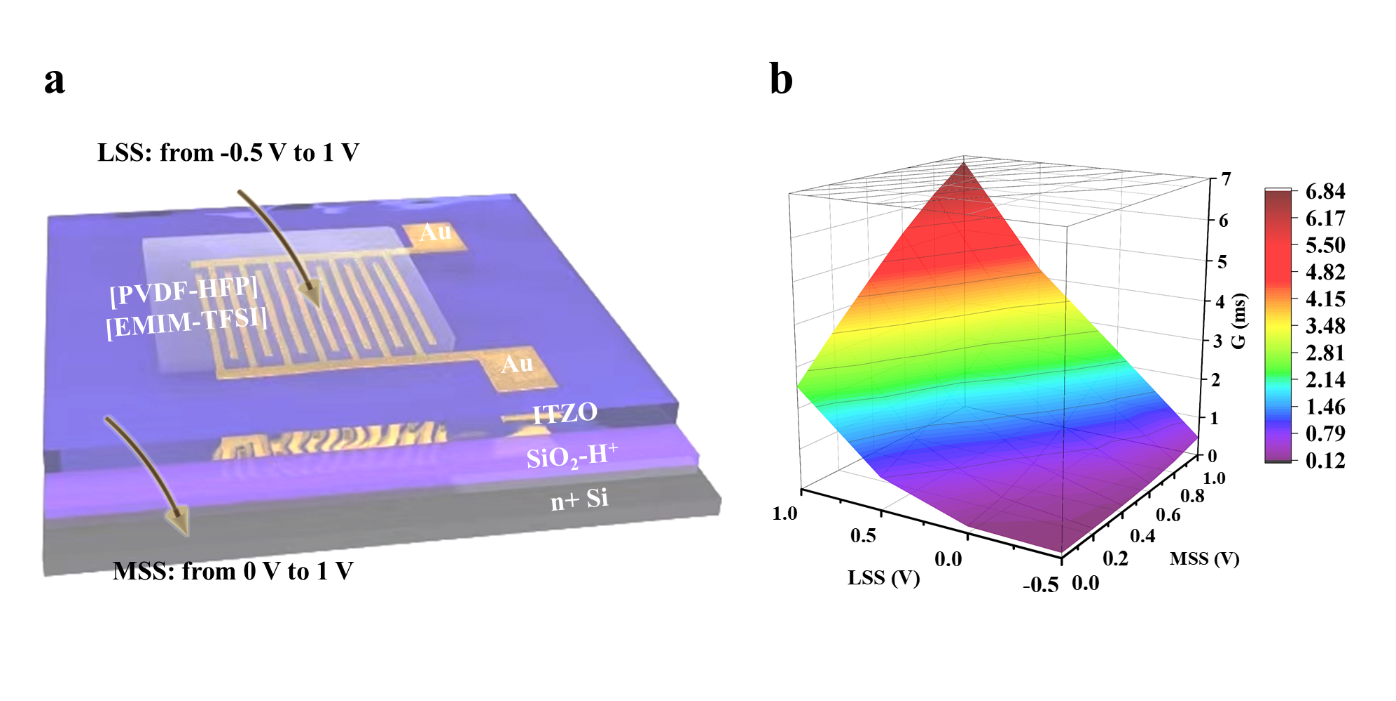
**

**Figure S2.** a) Schematic diagram of the HRAS test structure. b) Modulation effects of MSS and LSS on conductance.


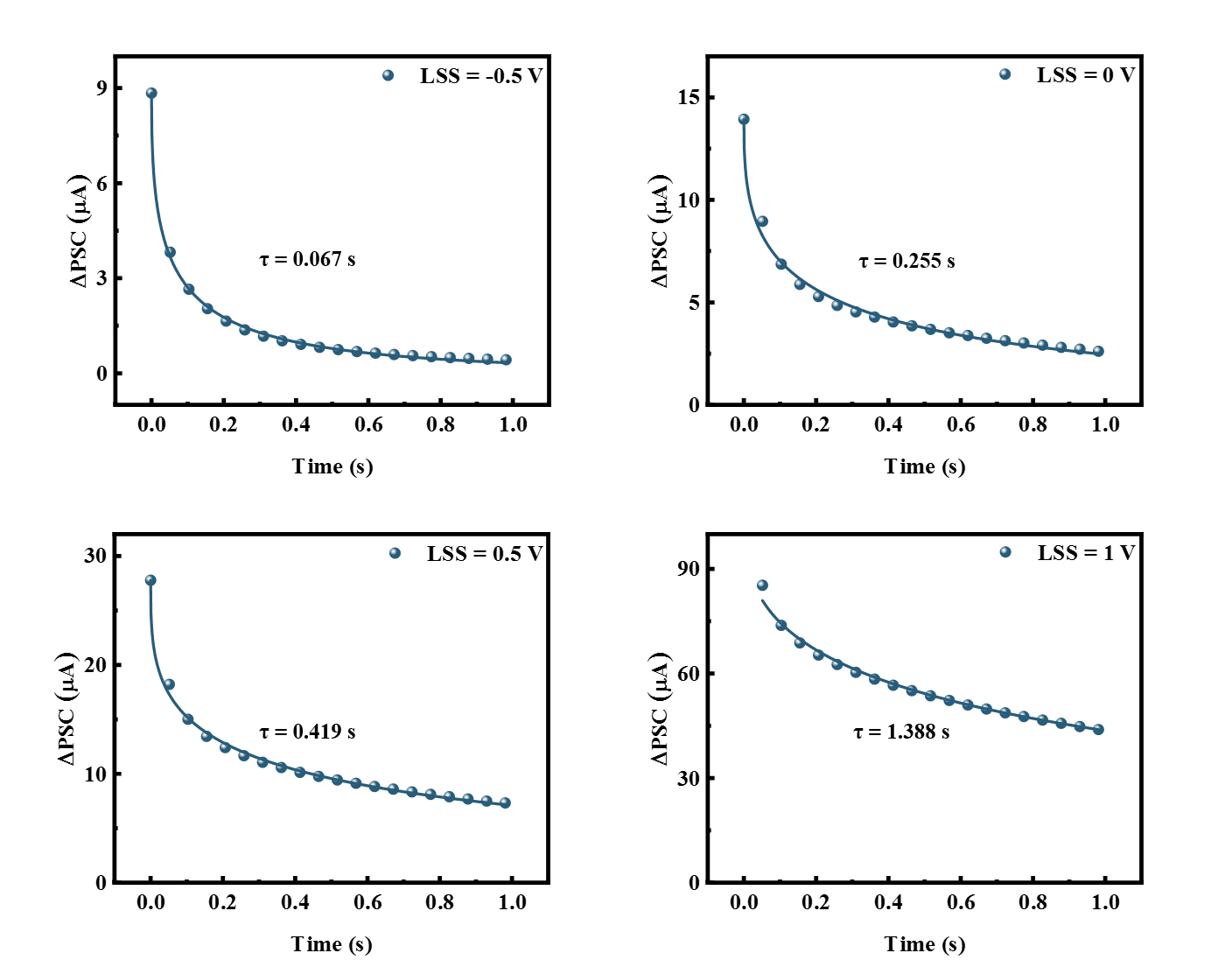


**Figure S3.** ΔPSC-retention curve after removing a single spike, under the coordinated control of different LSSs.

**Figure S4.** Comparison of transconductance parameters of HRAS devices with similar devices**.**


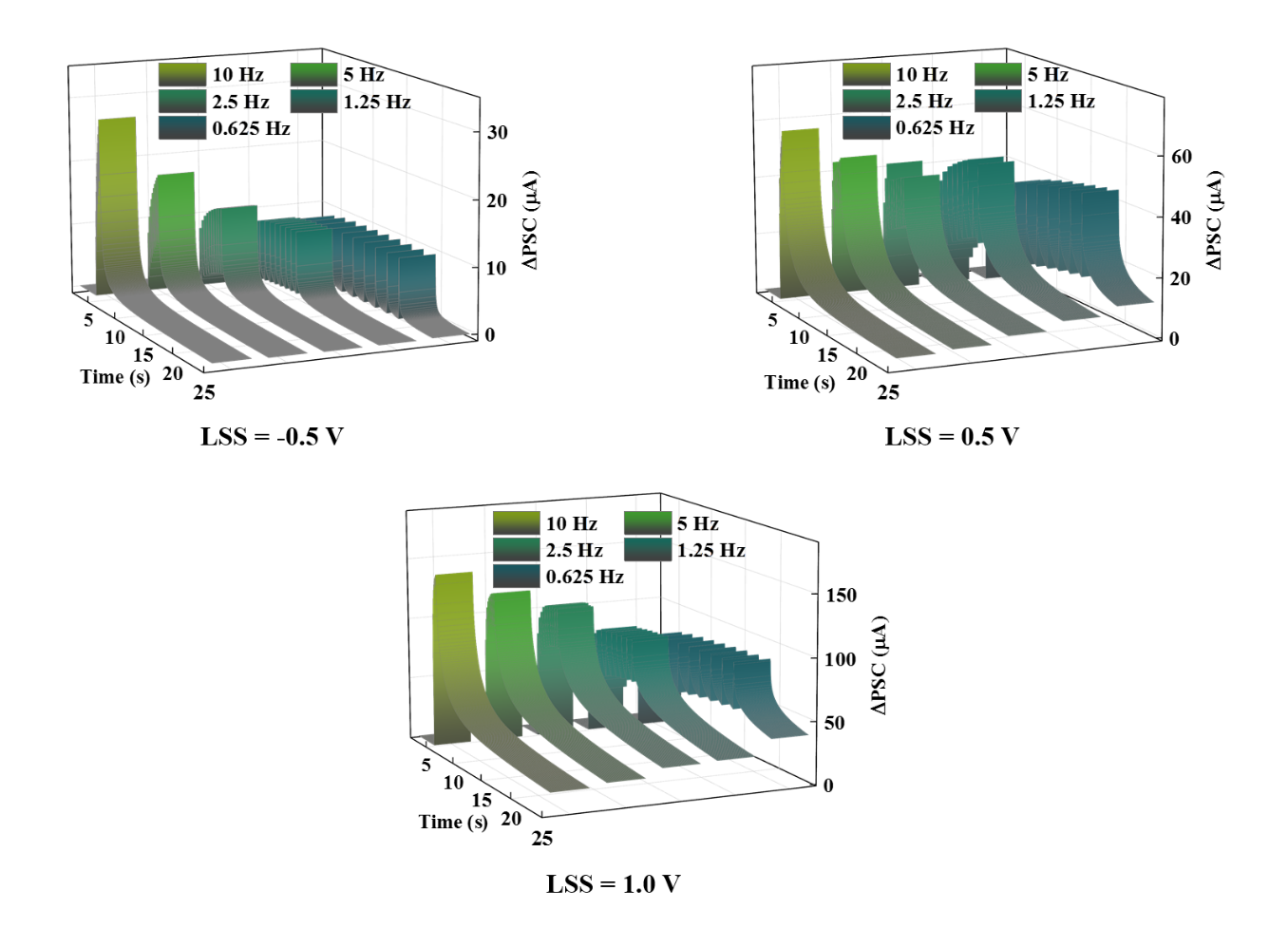


**Figure S5.** ΔPSCs trigged by a series of MSSs of different frequencies, under the coordinated control of different LSSs.


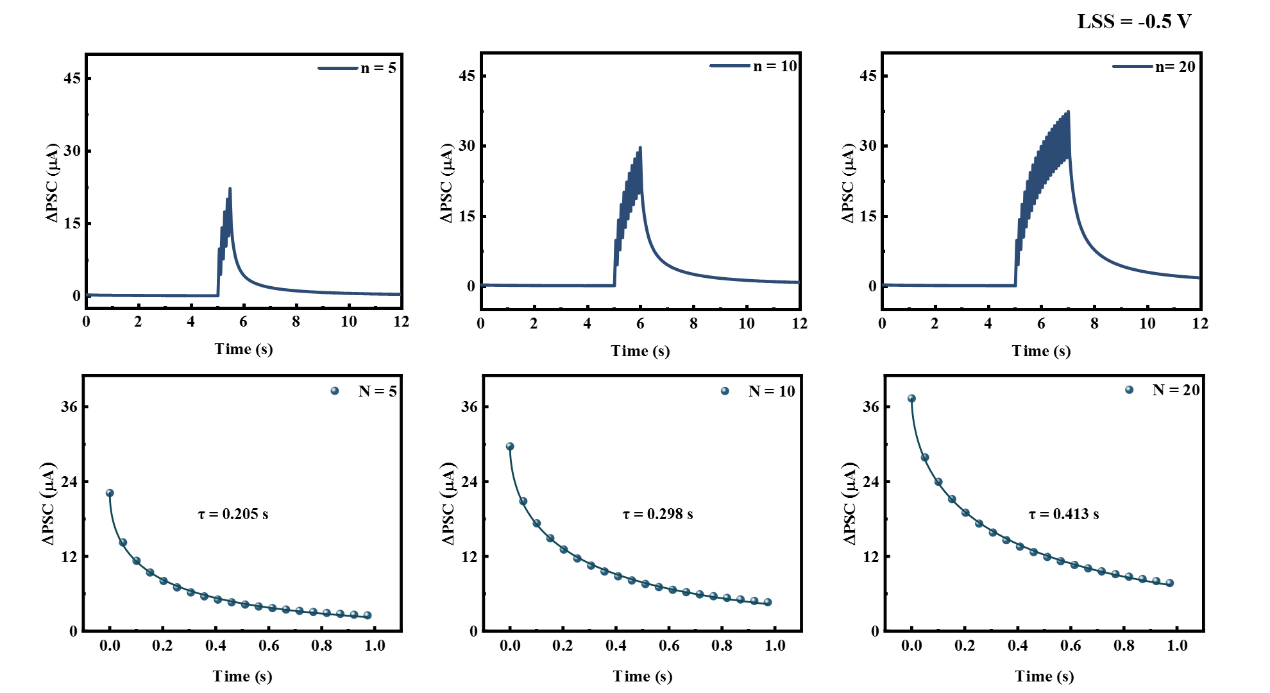


**Figure S6.** ΔPSCs trigged by a series of MSSs of different numbers and the ΔPSC-retention curve after removing the spikes, under the coordinated control of LSS = -0.5 V.


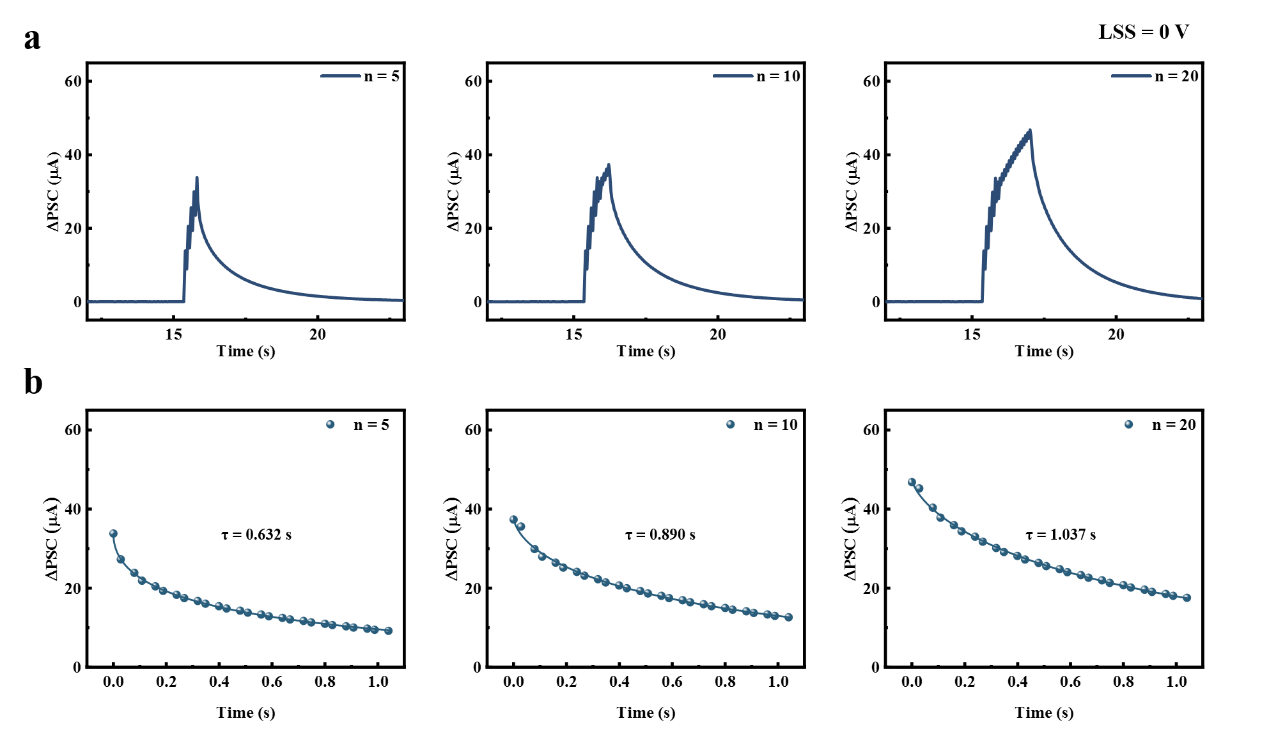


**Figure S7.** ΔPSCs trigged by a series of MSSs of different numbers and the ΔPSC-retention curve after removing the spikes, without the coordinated control of LSS.


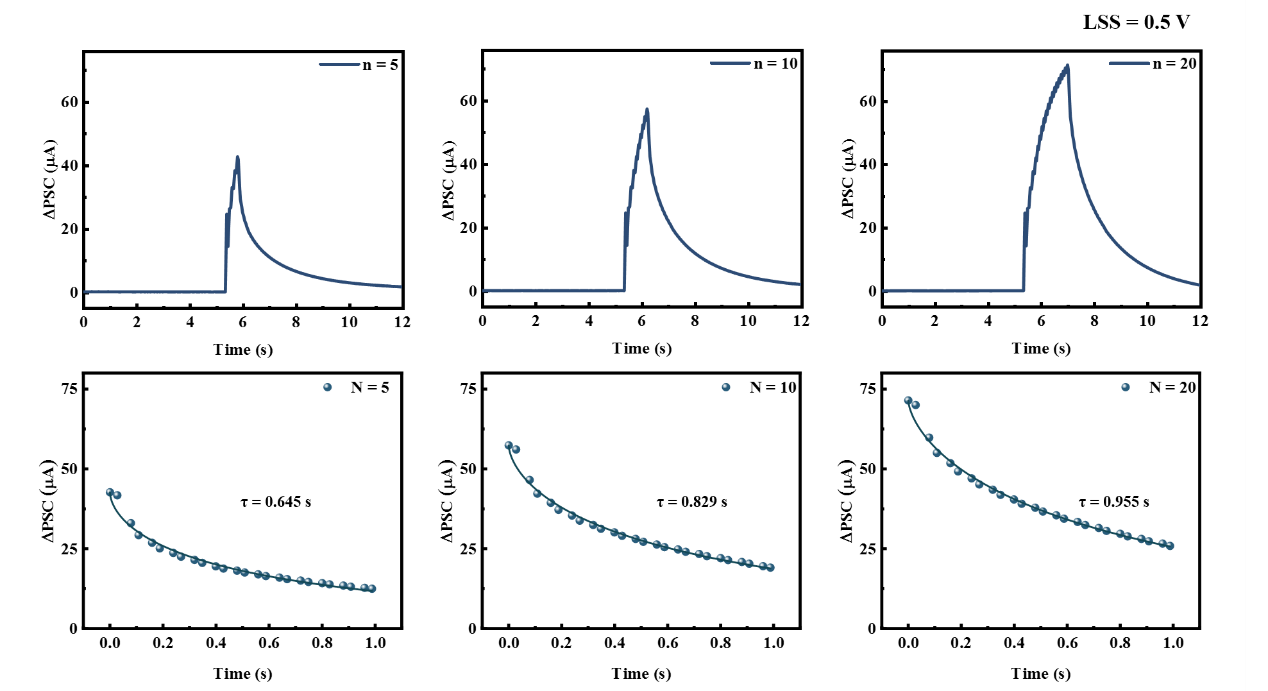


**Figure S8.** ΔPSCs trigged by a series of MSSs of different numbers and the ΔPSC-retention curve after removing the spikes, under the coordinated control of LSS = 0.5 V.


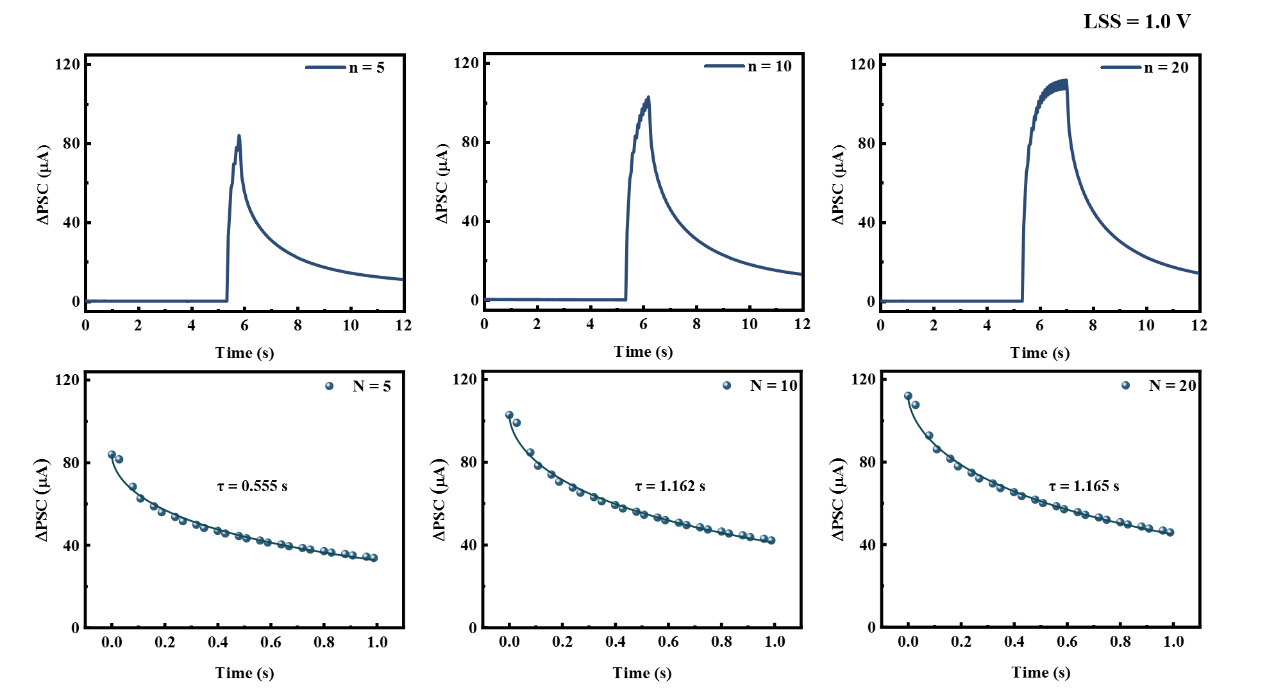


**Figure S9.** ΔPSCs trigged by a series of MSSs of different numbers and the ΔPSC-retention curve after removing the spikes, under the coordinated control of LSS = 1 V.


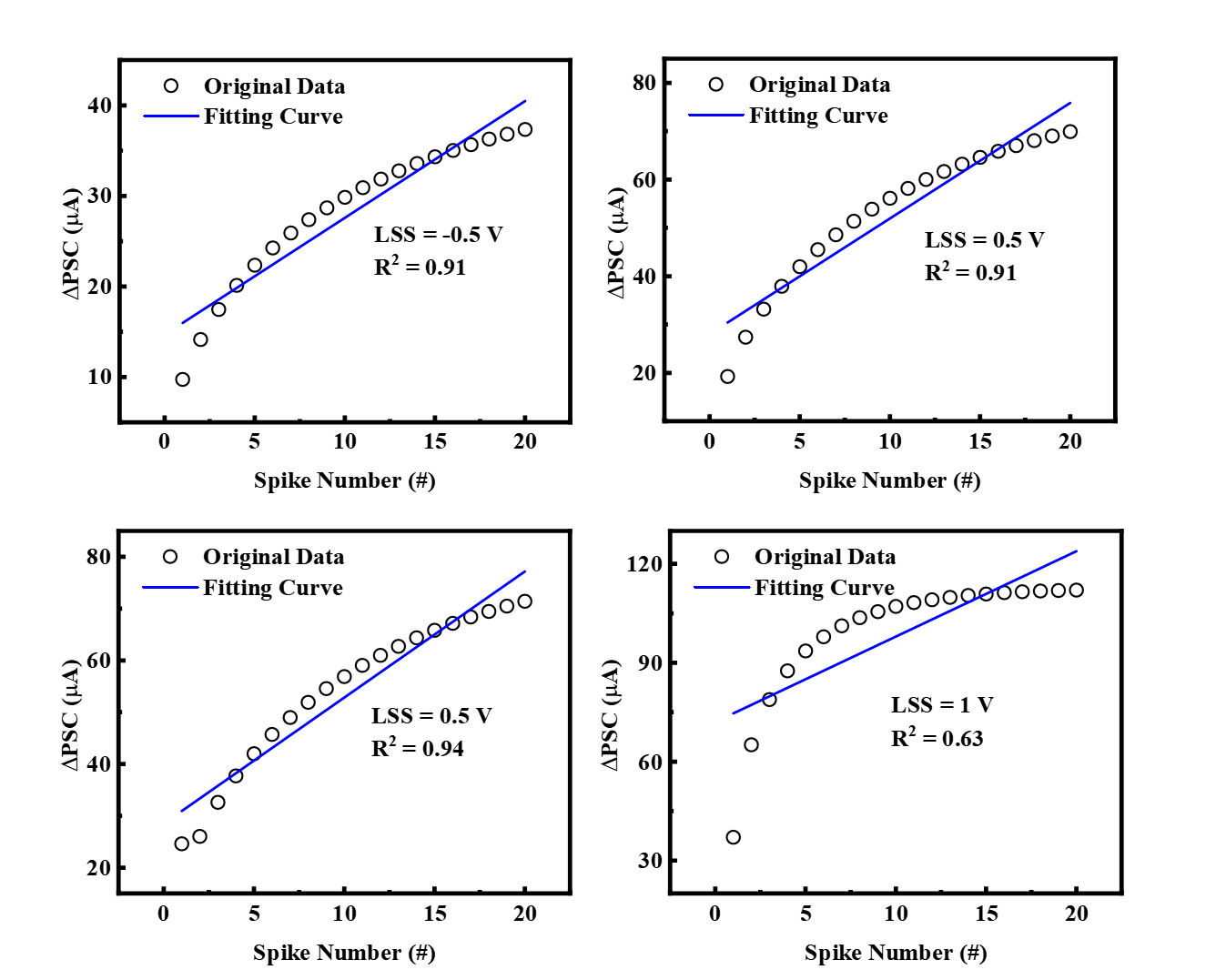


**Figure S10.** Fitting the linearity between PSC and pulse number in SNDP tests.


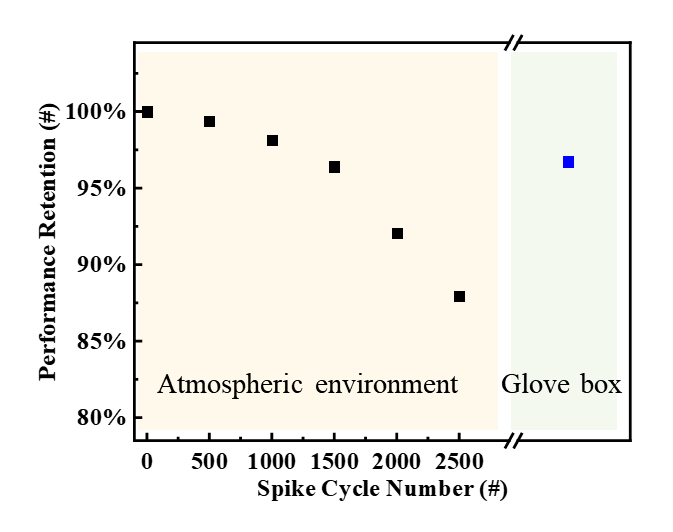


**Figure S11.** Reliability Evaluation of Device Performance under spike cycle number.


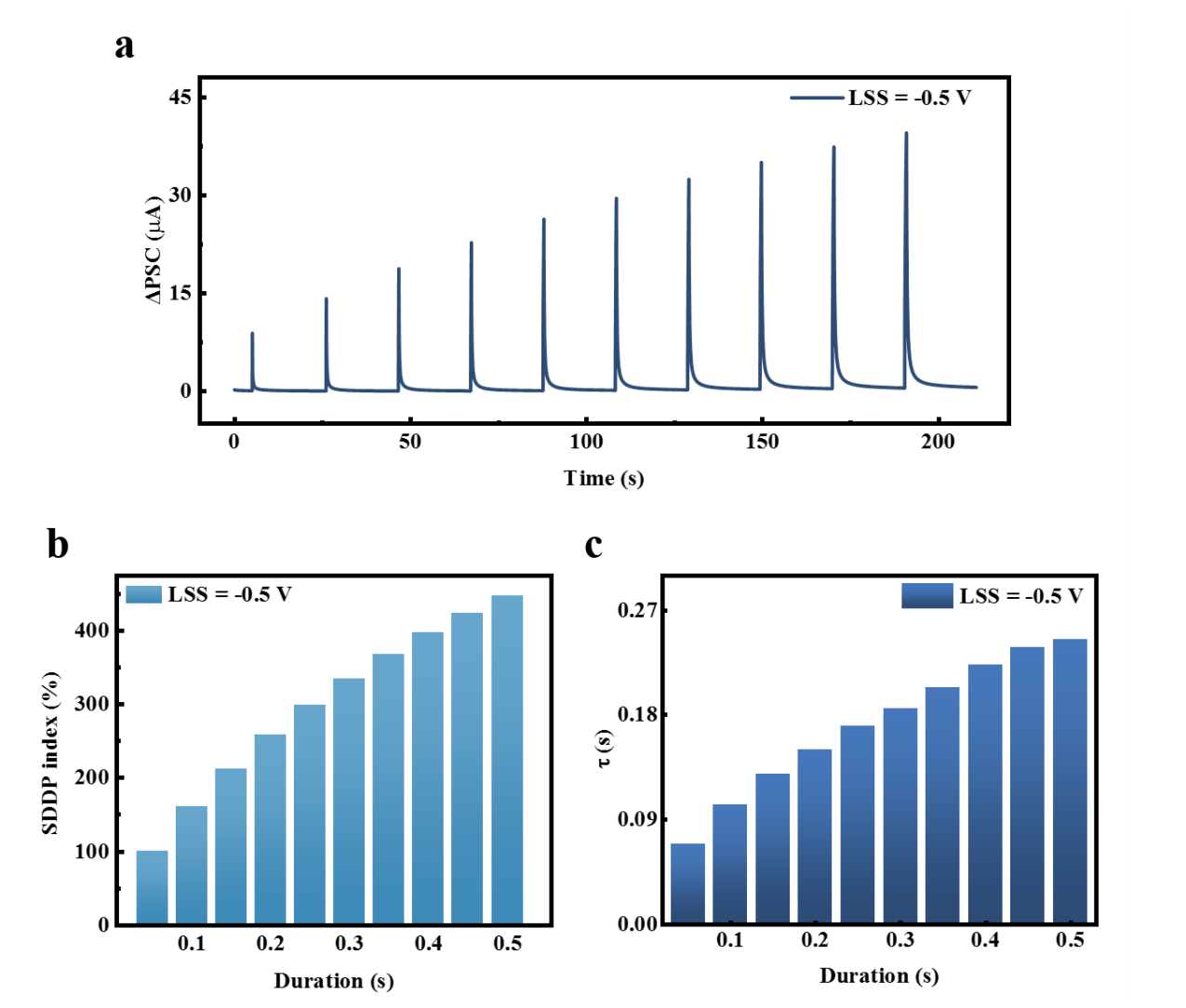


**Figure S12.** a) ΔPSCs trigged by a series of MSSs of different durations, under the coordinated control of LSS = -0.5 V. b) SDDP indexes (A_d_/A_0.05_ × 100%) trigged by a series of MSSs of different durations, under the coordinated control of LSS = -0.5 V. c) The fitting *τ* after removing spikes of different durations, under the coordinated control of LSS = -0.5 V.


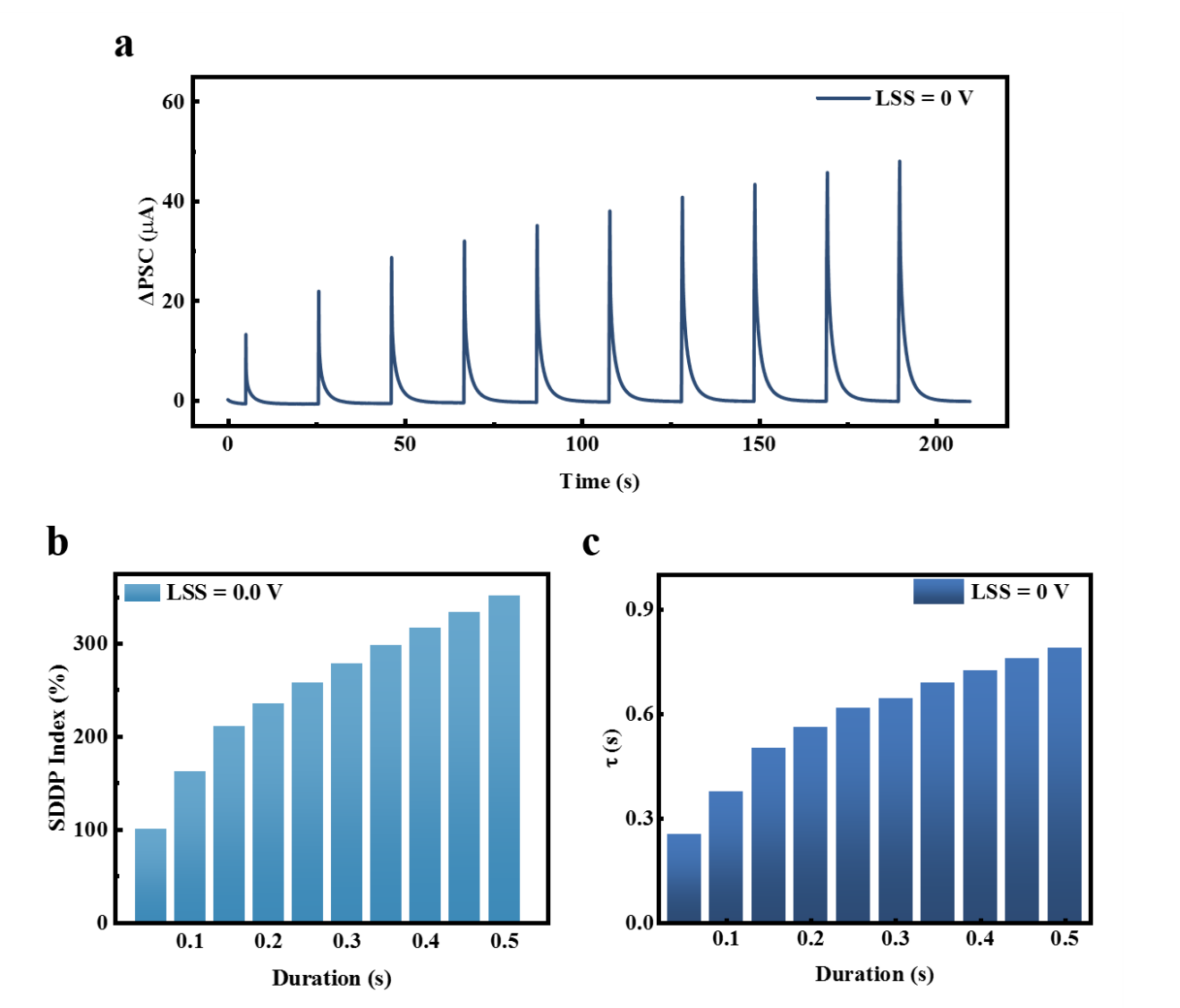


**Figure S13.** a) ΔPSCs trigged by a series of MSSs of different durations, without the coordinated control of LSS. b) SDDP indexes trigged by a series of MSSs of different durations, without the coordinated control of LSS. c) The fitting *τ* after removing spikes of different durations, without the coordinated control of LSS.


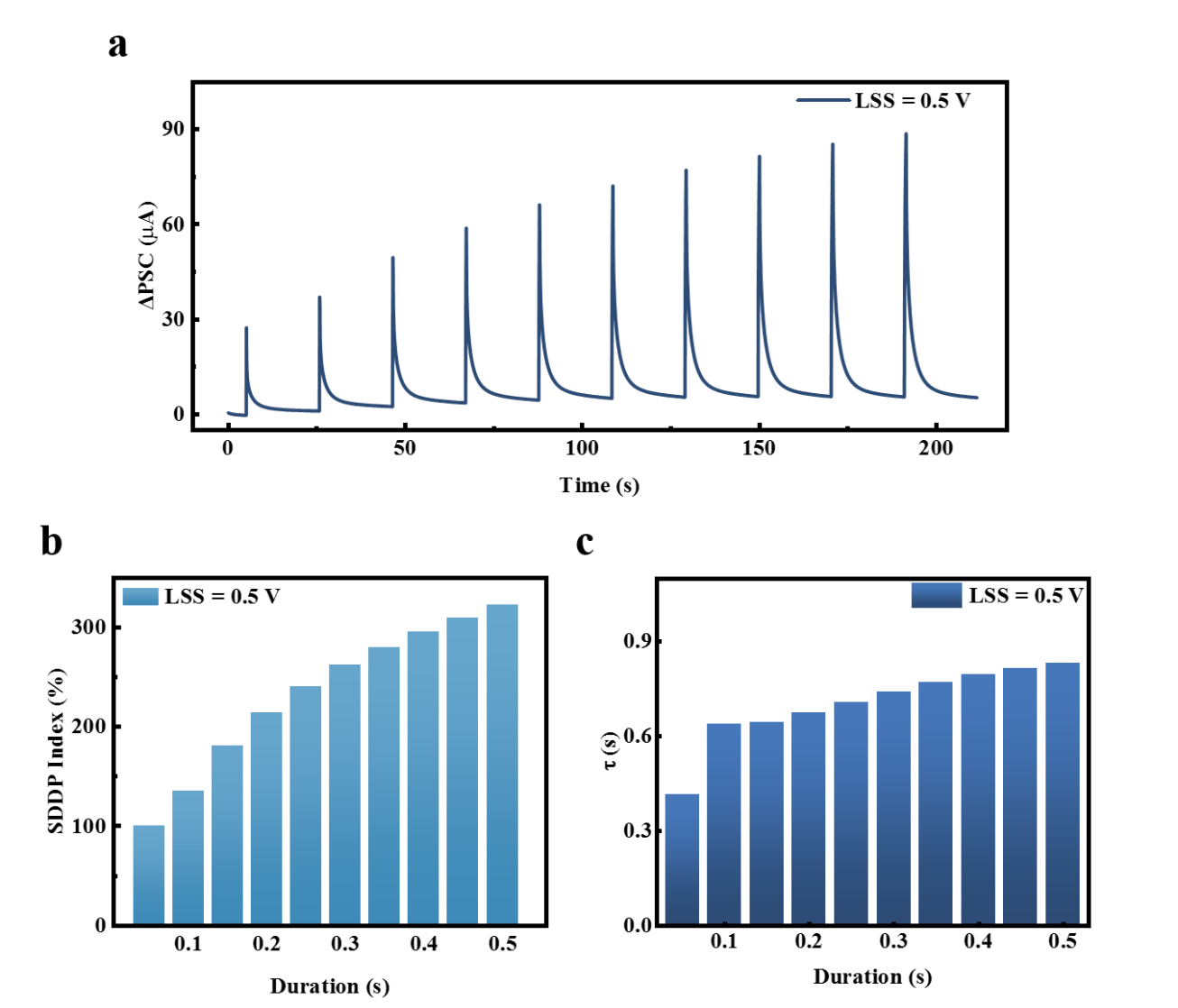


**Figure S14.** a) ΔPSCs trigged by a series of MSSs of different durations, under the coordinated control of LSS = 0.5 V. b) SDDP indexes trigged by a series of MSSs of different durations, under the coordinated control of LSS = 0.5 V. c) The fitting *τ* after removing spikes of different durations, under the coordinated control of LSS = 0.5 V.


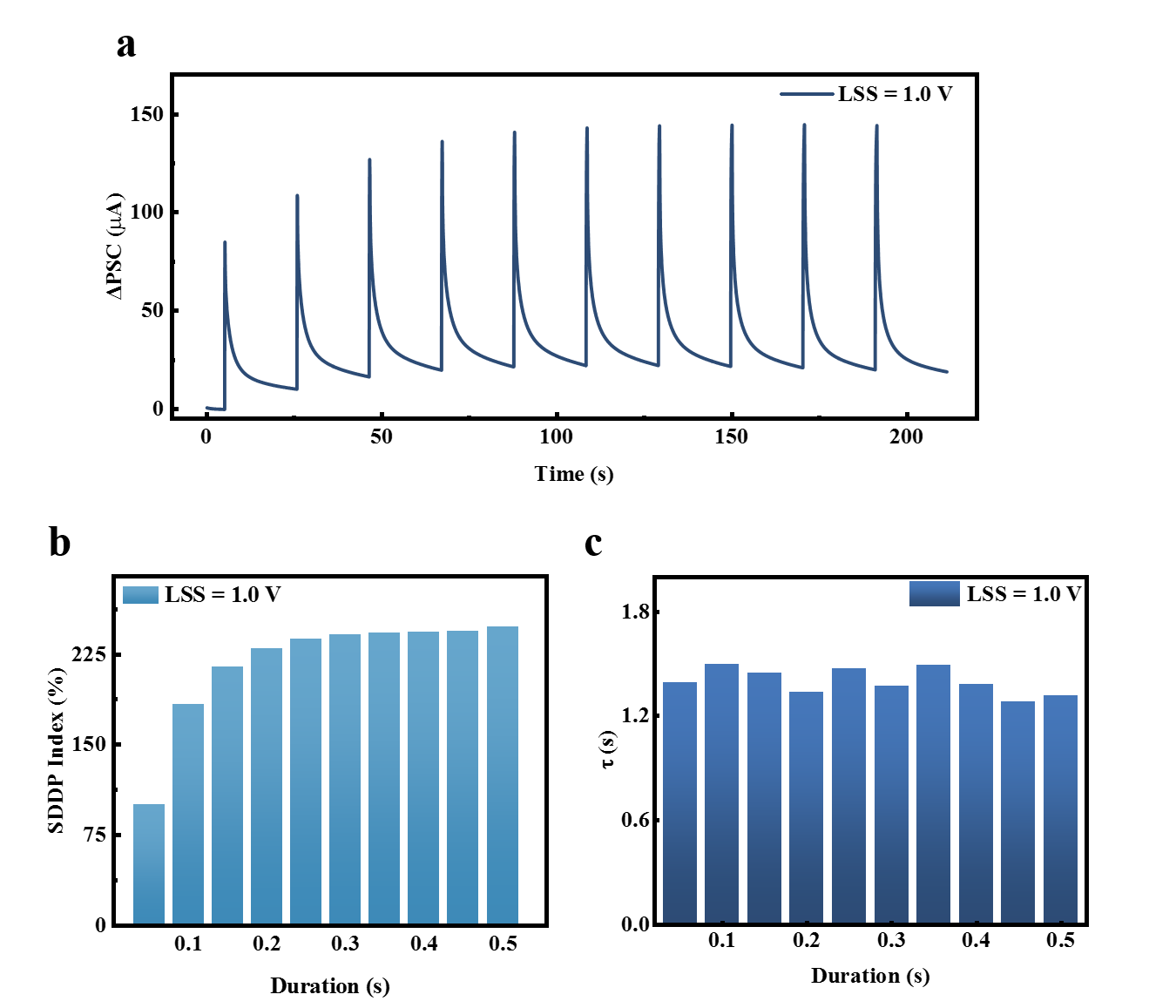


**Figure S15.** a) ΔPSCs trigged by a series of MSSs of different durations, under the coordinated control of LSS = 1 V. b) SDDP indexes trigged by a series of MSSs of different durations, under the coordinated control of LSS = 1 V. c) The fitting *τ* after removing spikes of different durations, under the coordinated control of LSS = 1 V.


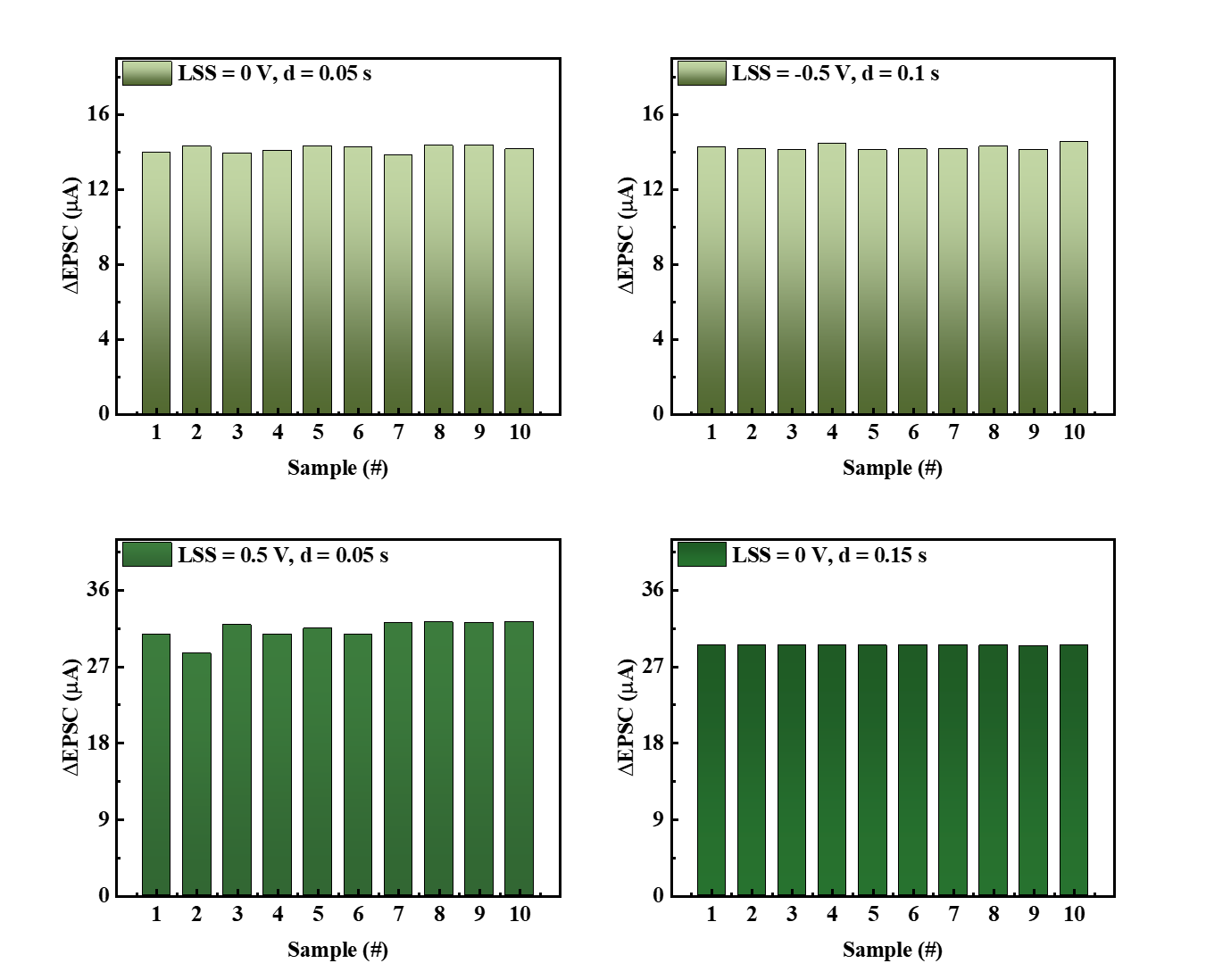


**Figure S16.** EPSC of 10 additional control samples for parallel validation.

**Table S1.** Comparison of this work with previous reports on artificial synapses that mimic various neurotransmitters with complex plasticity.

| **Channel Type** | **Channel materials** | **Material**  **properties** | **Mechanism** | **Synaptic features** | **Neuro-transmitters** | **Neural motif** | **Biological**  **applications** | **Ref** |
| --- | --- | --- | --- | --- | --- | --- | --- | --- |
| Dual channels | BP/SnSe | Unipolar | Charge trap | P/D regulation | Glu/GABA | × | × | [37] |
| Dual channels | P3HT/  TiO_2_ | Unipolar | Band engineering | LTP/STP switching | Glu/ACh | × | Simulate the brain's response to food intake | [12] |
| Dual channels | F_16_CuPc/  C8-BTBT | Unipolar | Exciton dissociation and Ion-dynamic | LTP/STP switching | DA/NA | × | Heart monitoring and alarming artificial nervous system | [38] |
| Dual channels | C8-BTBT/  ZnO | Unipolar | Ion-dynamic | P/D switching  LTP/STP switching | Glu/ACh | × | Simulation of hand withdrawal reflex and muscle memory | [13] |
| Dual channels | P3HT/  MoS_2_ | Unipolar | Ion-dynamic | P/D switching  LTP/STP switching | DA/NA | × | Simulation of Atkinson's achievement motivation | [39] |
| Dual channels | P3HT/  MoS_2_ | Unipolar | Ion-dynamic | P/D switching  LTP/STP switching | ACh/NA | × | An artificial autonomic nervous system | [40] |
| Single channel | PdSe_2_ | Ambipolar | Ion-dynamic | LTP/STP switching | Glu/DA | × | Logic signals backtrack | [24] |
| Single channel | Graphene | Ambipolar | Ion-dynamic | LTP/STP switching | Glu/ACh | × | Warning of negative emotions | [41] |
| Single channel | Graphene | Ambipolar | Charge trap | P/D switching | Glu/GABA | × | Artificial neuromuscular system for knee jerk reflex | [42] |
| Far-gate single channel | α-6T | Unipolar | Ion-charge dual-transfer mechanism | Enhanced memory /STP switching | Glu/ACh | × | Neurotransmitter-multiplexing temporal coding | [43] |
| Dual-gate heterointerface | ITZO | Unipolar | Coordination of ion-charge dual interfaces | 1. P/D switching with lateral modulations  2. LTP/STP multi-level intertwined plasticity | Glu/GABA | Lateral enhancement/  inhibition | 1. visual persistence with lateral modulations  2. Encryption and decryption with lateral modulations  3. Dual-bar input neural network structure for image recognition | This  work |

**Table S2.** Comparison of this work with previous reports relating to encrypted device.

| **Device types** | **Input stimuli** | **Channel type** | **Signal input mode** | **Temporal key** | **Spatial key** | **Neural motif** | **Approach of Encryption** | **Ref** |
| --- | --- | --- | --- | --- | --- | --- | --- | --- |
| Phototransistor | Light | Hetero-junction  thin film | Parallel  synchronous | × | × | × | UV-encoded encryption | [56] |
| Phototransistor | Light | 2D hetero-junction thin film | Parallel  synchronous | × | √ | × | UV-encoded encryption | [57] |
| Phototransistor | Polarized  light | Thin film | Parallel  synchronous | × | √ | × | Polarized light intensity encryption | [58] |
| Phototransistor | Light/  voltage | 2D hetero-junction thin film | Parallel  synchronous | × | √ | × | XOR logic gate encryption | [59] |
| Optoelectronic memristor | Light | Thin film | Sequential  asynchronous | × | × | × | Four-color light encoded  encryption | [60] |
| Optoelectronic memristor | Light | Homo-junction thin film | Sequential  asynchronous | × | × | × | Photoconductive encryption | [61] |
| Memristor | Voltage | heterojunction | Sequential  asynchronous | × | × | × | XOR logic gate encryption | [62] |
| Memristor | Voltage | Thin film | Sequential  asynchronous | × | × | × | XOR logic gate encryption | [63] |
| Transistor | Voltage | Thin film | Parallel  synchronous | × | √ | × | XOR logic gate encryption | [64] |
| Artificial synapse | Voltage | Thin film heterointerface | Parallel  synchronous | √ | √ | Lateral enhancement/  inhibition | Synaptic spike modulation with lateral modulations | This  work |
